# Structural and functional studies of Stf76 from the *Sulfolobus islandicus* plasmid–virus pSSVx: a novel peculiar member of the winged helix–turn–helix transcription factor family

**DOI:** 10.1093/nar/gku215

**Published:** 2014-03-25

**Authors:** Patrizia Contursi, Biancamaria Farina, Luciano Pirone, Salvatore Fusco, Luigi Russo, Simonetta Bartolucci, Roberto Fattorusso, Emilia Pedone

**Affiliations:** 1 Dipartimento di Biologia, Università degli Studi di Napoli “Federico II”, Napoli 80126, Italy; 2 Interuniversity Centre for Research on Bioactive Peptides (CIRPEB), University of Naples Federico II, Via Mezzocannone 16, 80134 Naples, Italy; 3 Istituto di Cristallografia, C.N.R., Bari 70126, Italy; 4 Dipartimento di Scienze e Tecnologie Ambientali, Biologiche e Farmaceutiche, Seconda Università di Napoli, Caserta 81100, Italy; 5 Istituto di Biostrutture e Bioimmagini, C.N.R., Napoli 80134, Italy

## Abstract

The hybrid plasmid–virus pSSVx from *Sulfolobus islandicus* presents an open reading frame encoding a 76 amino acid protein, namely Stf76, that does not show significant sequence homology with any protein with known 3D structure. The recombinant protein recognizes specifically two DNA-binding sites located in its own promoter, thus suggesting an auto-regulated role of its expression. Circular dichroism, spectrofluorimetric, light scattering and isothermal titration calorimetry experiments indicated a 2:1 molar ratio (protein:DNA) upon binding to the DNA target containing a single site. Furthermore, the solution structure of Stf76, determined by nuclear magnetic resonance (NMR) using chemical shift Rosetta software, has shown that the protein assumes a winged helix–turn–helix fold. NMR chemical shift perturbation analysis has been performed for the identification of the residues responsible for DNA interaction. In addition, a model of the Stf76–DNA complex has been built using as template a structurally related homolog.

## INTRODUCTION

Studies on crenarchaeal viruses have shown that they possess unique morphological features and quasi-orphan genome sequences that distinguish them from bacteriophages and eukaryal viruses (1–3). Indeed, the vast majority of genes from archaeal viruses do not have detectable homologues in the databases other than in other hyperthermophilic viruses (4). Seven families of double-stranded DNA viruses have been identified, among which the *Fuselloviridae* and *Rudiviridae* are the most well-studied specimens and therefore represent model systems for detailed studies of archaeal virus biology (5). These are indeed easily maintained under laboratory conditions and can be obtained in sufficient yields, which is not the case for many other archaeal viruses (6–10). A relatively high proportion of archaeal viral sequences is predicted to carry folds associated to transcription factor (TF) (11). The abundance of putative TFs probably reflects the importance of transcription regulation in the viral life cycle. As in the case of their hosts, the majority of the predicted TFs are bacterial-like and display ribbon–helix–helix, helix–turn–helix (HTH) or looped-hinge helix folds. In addition to bacterial-like TFs, archaeal viruses typically bear one or two sequences with Zinc Finger motifs (4). To date, structural and functional information on archaeal transcription regulators are scarce and only for few of them the 3D structure has been determined (6,8,12–14).

Two distinct genetic elements, SSV2 and pSSVx, belong to *Fuselloviridae* and coexist in the same *Sulfolobus islandicus* REY15/4 host, thus representing one of the few known two-virus systems in Archaea (15). pSSVx is a satellite virus that generates virus particles with the help of SSV2-associated packaging mechanisms. The transcriptional pattern of pSSVx undergoes a temporal variation of gene expression during its own life cycle, thus providing a good model for studying regulation of gene expression in Archaea (16,17). This genetic element encodes four TFs possibly implicated in the regulation of gene expression, i.e. ORF-c68, ORF51, ORF91 and ORF76. Among these, ORF76, here named Stf76 (*Sulfolobus* TF 76 amino acid protein), has homologs in almost all conjugative and cryptic plasmids from *Sulfolobus* (18), thus suggesting a relevant role for this protein in replication and/or maintenance of the plasmid.

Previous studies showed that transcriptional levels of the *Stf76* gene were constant and fairly high, similar to its homolog ORF80 from the pRN1 plasmid, whose DNA-binding capability has been described (19,20). Nevertheless, unlike ORF80 mRNA, which is synthesized at constant levels up to the stationary/death phase of the host growth (21), the expression of Stf76 transcript (T_stf76_) was completely inhibited during the process of pSSVx replication induction (16).

In this study, we have performed a detailed structural and functional characterization of Stf76. The corresponding gene has been cloned, expressed in *Escherichia coli* and the recombinant protein purified to homogeneity. To elucidate its interaction with the identified DNA operator sequence, analyses regarding its DNA-binding capabilities by means of electrophoretic mobility shift assay (EMSA), circular dichroism (CD), spectrofluorimetric and isothermal titration calorimetry (ITC) experiments have been performed. Moreover, a structural study has been undertaken by nuclear magnetic resonance (NMR) spectroscopy leading to (i) the solution structure of Stf76 based on CS-Rosetta approach (22,23), (ii) the characterization of the Stf76–DNA interaction by chemical shift perturbation (CSP) analysis, (iii) a structural model describing the interaction of a single Stf76 monomer with its DNA operator. Altogether these results contribute to elucidate the regulatory mechanism underpinning the role of this protein.

## MATERIALS AND METHODS

### Cloning, expression and purification of Stf76

The *Stf76* gene was polymerase chain reaction (PCR)-amplified from the plasmid pSSVx (cloned in pUC18) by using the primers Stf76fw 5'-CCCTATTTAACATATGGAAAAGGCGAAAC-3' and Stf76rv 5'-CATTACCCCGCTCGAGGTCGGCTAATTCATCTC-3'. The PCR product was digested with *Nde*I and *Xho*I and ligated to the pET-30a(+) digested with same restriction enzymes. Overexpression of Stf76 in the *E. coli* BL21-CodonPlus®(DE3)-RIL cells was induced at OD_600nm_=0.8 by the addition of 0.5 mM IPTG (isopropyl β-D thiogalactopyranoside) for 16 h. The cells from 1 l of culture were resuspended in 20 ml of lysis buffer (50 mM sodium phosphate buffer, 300 mM NaCl pH 7.0) containing complete protease inhibitor cocktail tablets (Roche). The crude extract was subjected to heat treatment at 70°C for 20 min and then centrifuged for 15 min. The extract was applied to a HisTrap HP (GE Healthcare) equilibrated with buffer A (20 mM sodium phosphate, 300 mM NaCl, 10 mM imidazole pH 7.0). The column was washed with buffer A plus 30 mM imidazole, proteins were eluted with the same buffer A supplemented with 250 mM imidazole. Protein-containing fractions were pooled and loaded on to a Superdex 75 16/60 column (GE Healthcare) and the elution was carried out in 20 mM sodium phosphate buffer (pH 7.0) containing 150 mM NaCl. Finally, Stf76 was dialyzed overnight against 10 mM sodium phosphate buffer, 50 mM NaCl pH 7.0.

The concentration of the purified protein was measured using the theoretical extinction coefficient ϵ = 10 095 M^−1^ cm^−1^ at a Nanodrop 2000 Spectrophotometer (ThermoScientific). Alternatively, the Pierce BCA Protein Assay or Coomassie (Bradford) Protein Assay were utilized.

### EMSA assay

Binding of Stf76 to target DNA sequences was assayed by EMSA experiments. Two distinct DNA regions (herein named site A and site B), located in the promoter of the *Stf76* gene and previously indicated as putative binding sites of Stf76 (19), were analyzed. The 137 bp DNA fragment encompassing both sites A and B (probe A+B) was amplified by PCR using the pSSVrt (24) as template and the following primers 5′-Stf76 siteA/B (5′-GTTAGCCCACGCGTGAAGGGAAA-3′) and 3′-Stf76 pr siteA/B (5′-AGTTTCGCCTTTTCCATACGTTAAATAGGG-3′). In the displacement experiments, the binding reactions were performed with 3.2 μM Stf76 with the concurrent addition to the EMSA mixtures of increasing amounts of specific unlabeled probe (1:10, 1:100, 1:500, 1:1000 ratio of labeled/unlabeled specific DNA) or Salmon Sperm DNA as aspecific competitor (1:1000, 1:2000 ratio of labeled specific/aspecific DNA).

In order to analyze the binding of Stf76 to each of the two interacting regions, a sequence of 52 bp including only site A (probe A) was PCR-amplified using the following primers 5′-Stf76 siteA/B and Revplr1A(5’-TTCGCCTTCTGAAAATTTGTCTTCATAACAC-3’) and used in EMSA assay in comparison with a probe encompassing site B (probe B, 48 bp) obtained with the following primers couple 5’-siteB (5’-GTGTTATGAAGACAAATTTTCAGAAGGCGAA-3’)/3′Stf76 siteA/B. A shorter sequence, retaining specific binding of Stf76 to site A, was obtained by annealing a 35-nt-long ^32^P-labeled oligodeoxynucleotide (probe A’) encompassing site A (5′-GGAAACAGTATTAATAAAGTGTTAATCCTATTACCC-3′) with a complementary oligodeoxynucleotide in a buffer containing 10 mM Tris-HCl pH 7.5, 50 mM NaCl and 1 mM EDTA. Finally, a mutated version of the oligodeoxynucleotide probe A’ (probe A’* 5’-GGAAACAGTATTAATAAAGTGCCGTTCCTATTACC-3’) was employed to analyze the binding capability of Stf76 to site A.

All the EMSA assays were conducted by a thermal pre-incubation of the purified Stf76 protein for 15 min at 50°C in assay buffer [20 mM Tris-acetate (pH 8.0), 50 mM potassium acetate, 10 mM magnesium acetate, 1 mM DTT (dithiothreitol) and 5% (v/v) glycerol] in the presence of 1 μg of Salmon Sperm DNA as aspecific competitor and by adding the labeled probes at a concentration of 5–10 nM. The binding reactions were performed with increasing amounts of Stf76 (3.2, 6.4, 9.6, 12.8, 16, 19.2, 22.4, 25.8, 32.4, 40 and 51.6 μM) for 30 min at 37°C and analyzed on 6% or 10% polyacrilamide gels (depending on the size of the probe) in 0.5× TBE.

Gels were transferred onto filter paper, dried and revealed both by Molecular Dynamics Bio-Rad PhosphorImager and/or autoradiography.

### CD

Far-UV CD spectra (260–190 nm) were recorded by using a Jasco J-715 spectropolarimeter, equipped with a PTC-423S/15 peltier temperature controller. Spectra were acquired at 20°C according to the following parameters: band width of 1 nm, response of 8 s, data pitch of 0.2 nm and scanning speed of 10 nm/min. CD measurements were carried out using a 0.1-cm-pathlength cell and a protein concentration of 10 μM in a 10 mM sodium phosphate, 50 mM NaCl pH 7.0 buffer. For the titration with the DNA, concentration of the double-stranded oligonucleotides (35 bp-site A) was varied from 0 to 10 μM. The baselines were corrected by subtracting buffer and DNA spectra.

### Fluorescence

Stf76 at final concentration of 5 μM in 10 mM sodium phosphate, 50 mM NaCl pH 7.0 buffer was excited at 295 nm and emission spectra among 290 and 450 nm were measured by a spectrofluorimeter (Varian). The spectra were registered also in the presence of increasing concentration of the double-stranded oligonucleotides (35 bp-site A) from 0 to 10 μM.

### Thermal denaturation of DNA

Double-stranded DNA (35 bp-site A) at a concentration of 5 μM was incubated in 10 mM sodium phosphate, 50 mM NaCl pH 7.0 with and without 10 μM Stf76 in a 0.5 ml cuvette. The absorbance at 260 nm was monitored while the cuvette was heated with a peltier element from 20 to 80°C, with an increment of temperature of 1°C/min.

### Cross-linking

Stf76 (10 μM) was incubated with and without 10 μM double-stranded oligonucleotides (35 bp-site A) in the presence of 100 μM of Bis(sulfosuccinimidyl) suberate (BS3) in 10 mM sodium phosphate, 50 mM NaCl pH 7.0 buffer, for 1 h at room temperature in a reaction volume of 20 μl. The samples were separated on 18% sodium dodecyl sulphate-polyacrylamide gel electrophoresis (SDS-PAGE).

### Dynamic light scattering and static light scattering

Size measurements of 50 μM Stf76 were performed on a Nano Zetasizer, spectrometer (Malvern, UK) in 10 mM sodium phosphate, 50 mM NaCl pH 7.0 buffer. The wavelength of the laser was 632.8 nm, and the scattering angles 90° and 175°. For each sample, the mean value of particles' diameters was calculated from three replicate determinations. The molecular diameter is calculated from the autocorrelation function of the intensity of light scattered from the particles assuming a spherical form of particles. For molecular weight measurements, a MiniDAWN Treos spectrometer (Wyatt Instrument Technology Corp.) equipped with a laser operating at 658 nm was used connected on-line to a size-exclusion chromatography. Seventy micromolar Stf76 in 10 mM sodium phosphate, 100 mM NaCl pH 7.0 and 35 μM of double-stranded oligonucleotides (35 bp-site A) was loaded alone and in a molecular ratio of 1:1 and 2:1 on a WTC-015S5 (150 Å) column (Wyatt technology) and analyzed by size-exclusion chromatography connected to a triple-angle light scattering detector equipped with a QELS (Quasi-Elastic Light Scattering) module. A constant flow rate of 0.5 ml/min was applied. Elution profiles were detected by a Shodex interferometric refractometer and a mini Dawn TREOS light scattering system. Data were analyzed by using Astra 5.3.4.14 software (Wyatt Technology).

### ITC

ITC studies were performed at 22°C with an iTC200 calorimeter (MicroCal/GE Healthcare, Milano, Italy). Samples were extensively dialyzed in the same buffer (20 mM sodium phosphate, 50 mM NaCl pH 7.0) prior ITC measurements. A solution of Stf76 at a concentration of 100 μM was titrated into a 10 μM double-stranded oligonucleotide 35 bp-site A solution. To exclude the presence of artifacts due to ligand dilution ITC runs were performed by titrating of Stf76 into the buffer. Fitting of data to sequential binding sites model was carried out with the Origin software as supplied by GE Healthcare.

### Protein alkylation and LC-ESI-MS analyses

Stf76 (50 μg) was denatured in 50 μl of a solution containing 20 mM sodium phosphate, 6 M guanidinium chloride pH 7.0 for 1 h at 25°C, in the absence of reducing agents. The protein was then subjected to alkylation by incubation of the sample in 0.12 M 4-vinyl-pyridine (4-VP) (Sigma-Aldrich) at 25°C for 60 min, after which time the reaction was terminated by quenching at 4°C. As a control, after denaturation, the protein was reduced before 4-VP alkylation. Then 0.2 μg of alkylated and control sample were run on a Liquid Chromatography-Electrospray-Mass Spectrometry (LC-ESI-MS) instrument. The molecular mass of the protein was estimated using electrospray mass spectra recorded on a Bio-Q triple quadrupole instrument (Micromass, Thermofinnigan, San Jose, CA, USA) as described previously (25).

### NMR spectroscopy

All NMR experiments were carried out at 25°C using an Inova 600 MHz spectrometer (Varian Inc., Palo Alto, CA, USA), equipped with a cryogenic probe optimized for ^1^H detection.

^15^N/^13^C- and ^15^N-labeled samples were prepared through a procedure similar to that described above, but cells were grown in M9 medium with the addition of ^13^C-labeled glucose and ^15^N-labeled ammonium chloride (26).

Samples used for NMR structural studies contained ∼200 μM Stf76 in 20 mM sodium phosphate pH 5.5, 50 mM NaCl, 0.02% sodium azide and 10% ^2^H_2_O.

### Structure determination

Sequential backbone signal assignment was first carried out by combined analyses of the 3D triple-resonance spectra HNCA (27–29) and HN(CO)CA (29,30), and by evaluation of sequential nuclear Overhauser effects (NOEs) in a ^15^N-edited NOESY spectrum (31–33), acquired with a mixing time of 100 ms. Complete H^N^, N, Cα and Hα resonance assignments of all ^15^N-HSQC-detected residues were subsequently achieved based on ^15^N-edited TOCSY spectrum (31), acquired with a mixing time of 50 ms. Four glutamine and one asparagine side chain amides were detected and unambiguously assigned in the ^15^N-edited NOESY spectrum. The indolic HN group of the only Trp residue was also unambiguously assigned. All NMR data were processed with the software VNMRJ 1.1.D (Varian Inc.). 2D and 3D spectra were analyzed using tools available in CARA software (34).

Secondary structural elements of Stf76 were initially identified by chemical shift index (CSI) analysis (35), which was generated using Cα and Hα chemical shifts. Dihedral angle restraints were calculated from H^N^, Cα, Hα and N chemical shifts with the software TALOS+ (36).

The structure calculation was performed with the program CS-Rosetta (22,23) using as structural restraints the torsion angles ϕ/ψ derived from TALOS+ database and the H_N_, Cα, Hα and N chemical shifts of those residues indicated by TALOS+ to be rigid in pico-second timescale with an order parameter *S*^2^ > 0.7. A set of 200 fragment candidates matching these chemical shifts was used to calculate 3000 structures in Rosetta. The energy of these Rosetta structures was then rescored against the observed chemical shifts and the 20 conformers with the lowest rescored energy were selected for the ensemble. The structures were visualized and evaluated by using the programs MOLMOL (37), PROCHECK-NMR (38) and MOLPROBITY (39).

The most representative model of the Stf76 structure was compared to structures in the Protein Data Bank by using the DaliLite server (40).

^15^N relaxation parameters of Stf76 were evaluated by recording inversion recovery ^1^H-^15^N HSQC for R_1_ measurements and spin echo ^1^H-^15^N HSQC for R_2_ measurements. Acquisition parameters and relaxation data processing and analysis were essentially as reported (41,42). An estimate of the isotropic correlation time, τc, of Stf76 was obtained from the ratios of ^15^N R_2_ and R_1_ values of residues that passed the coarse and fine-filtering steps (43), using the equation reported (44). The Stokes–Einstein–Debye equation was then used to calculate the hydrodynamic radius from the τc. The hydrodynamic properties were also evaluated using the NMR software hydropro (45).

### NMR analysis of Stf76–DNA interaction

CSP studies of Stf76 with the 35-bp-site A sequence were carried out using ^15^N-labeled Stf76 dissolved in 500 μL at 100 μM concentration in 20 mM sodium phosphate pH 5.5, 50 mM NaCl, 0.02% sodium azide and 10% ^2^H_2_O. The DNA fragment, prepared as reported above, was first dissolved at 500 μM concentration in 20 mM sodium phosphate pH 5.5 buffer, 50 mM NaCl and then carefully mixed with Stf76 at a final concentration of 10 and 50 μM, as reported in Omicinsky *et al.* (46). 2D [^15^N, ^1^H] Heteronuclear Single Quantum Coherence (HSQC) spectra were acquired in the absence and presence of DNA. Starting from the amide resonances for Stf76 free, average combined chemical shift changes for Stf76 bound were determined using the following equation: ΔδHN_av_ = [((Δδ_H_)^2^+(Δδ_N_/5)^2^)/2]^1/2^, where Δδ_H_ and Δδ_N_ are the chemical shift variations of the amide proton and nitrogen resonances (47–50), respectively. Moreover, intensity reduction of the amide cross-peaks over one standard deviation was also taking in account to define the DNA-binding site.

## RESULTS

### Cloning, expression and purification of Stf76

The gene *Stf76* was cloned into the expression vector pET30a(+) and the recombinant protein was expressed in BL21(DE3) codon-plus ArglleLeu (RIL) as a soluble C-terminal His-tag fused protein. Stf76 was purified to homogeneity in a three-step procedure: the soluble fraction was heated at 70°C for 20 min taking advantage from the high thermostability of the protein, successively, an affinity and a size-exclusion chromatography allowed to obtain an homogeneous sample with a yield corresponding to ∼10 mg of protein per liter of culture.

**Figure 1.**
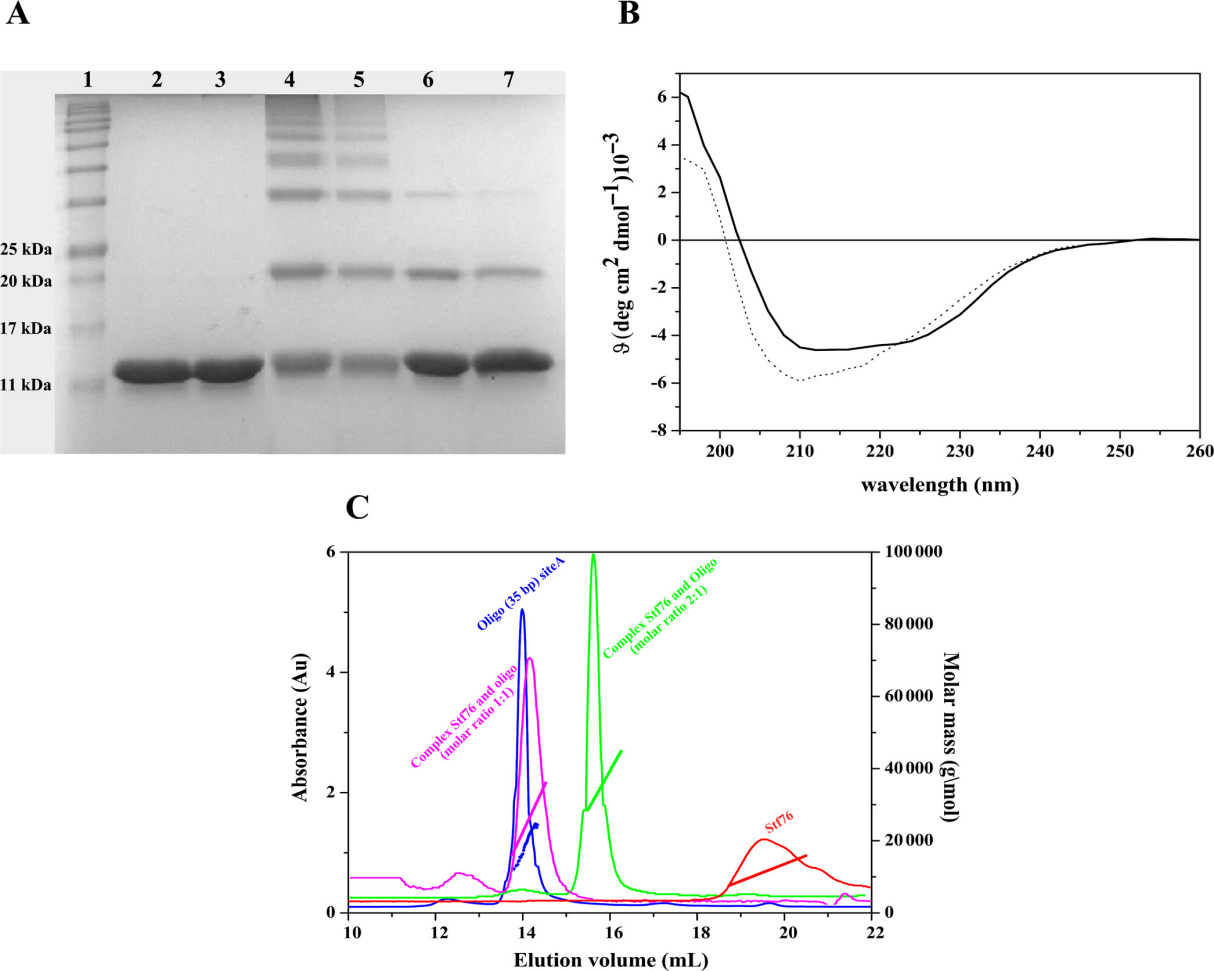
(**A**) Chemical cross-linking of Stf76 with BS3. Stf76 (10 μM) was incubated with and without cross-linker and analysed on SDS-PAGE. Lane 1: molecular weight markers; lane 2: recombinant Stf76; lane 3: Stf76 in the presence of DNA (10 μM); lanes 4 and 5: Stf76 in the presence of BS3 (100 μM and 200 μM, respectively); lanes 6 and 7: Stf76 in the presence of BS3 and 35 bp-site A. (**B**) Far-UV CD spectra of Stf76. CD spectra of Stf76 10 μM free (continuous line) and complexed with 5 μM of 35 bp-site A (dotted line). (**C**) Light-scattering measurements. A graph of the molecular mass and absorbance at 280 nm versus the elution volume. In red is reported Stf76; in blue 35 bp-site A; in purple the complex of Stf76 and 35 bp-site A (molar ratio 1:1), in green the complex of Stf76 and 35 bp-site A (molar ratio 2:1).

SDS/PAGE analysis showed that the purified protein migrated as a single band with an expected molecular mass of ∼10 kDa (Figure 1A, lane 2), which agrees with the theoretical (10281 Da was also taken into account the presence of the LEHHHHHH tag) as well as with the molecular mass determined by LC-SI-MS. Moreover, Stf76 contains a disulphide bridge that links cysteines 21 and 67, as obtained by LC-ESI-MS analysis (data not shown). However, the disulphide bridge seems to not affect the structure (see below) considering that CD and fluorescence spectra registered in the presence or not of a reducing agent did not show any differences (data not shown).

### CD studies

Far-UV CD spectrum of Stf76 measured at 20°C was characterized by two minima, at 208 and 222 nm and one maximum at <200 nm (Figure 1B, continuous line), in agreement with a properly folded protein. The two broad minima, reasonably affected by the presence of β elements, are features indicative of the presence of both α and β secondary structure elements, as confirmed by the deconvolution of CD data performed by using the software CDPro. In addition, the protein proved to be highly stable being not significantly affected by exposure to increasing temperatures until 100°C (results not shown).

### Analysis of the oligomerization state of Stf76 in solution

To assess Stf76 quaternary structure, chemical cross-linking experiments, size-exclusion chromatography coupled with a triple-angle light scattering-QELS and NMR were used.

A Stf76 tendency to oligomerize was observed in chemical cross-linking experiments performed with the homo-bifunctional amino-reactive reagent BS3. When Stf76 was incubated with BS3 and the reaction products were separated on a denaturing gel, multiple forms of Stf76 were found (Figure 1A, lanes 4 and 5).

Static light scattering analysis showed that Stf76 has a molecular mass of about 13 kDa, which indicated that the protein is prevalently a monomer in solution (Figure 1C, in red). Dynamic light scattering (DLS) analysis conducted at concentrations ranging from 50 μM to 300 μM, indicated that Stf76 shows a concentration-dependent monomer/oligomer equilibrium in the conditions assayed. Indeed, a single peak corresponding to a monomer appeared until to 200 μM, while increasing concentrations resulted in the formation of oligomers (data not shown).

Hydrodynamic properties of Stf76 in solution were estimated at 200 μM using NMR backbone ^15^N longitudinal (R_1_) and transverse (R_2_) relaxation rate measurements (Supplementary Figure S1) in combination with DLS and structure-derived data (41,42). An estimation of the isotropic rotational correlation time, τ_c_, was obtained from the R_2_/R_1_ ratio of the 54 residues that passed the coarse- and fine-filtering steps (43), yielding a mean ± SD value of 8.4 ± 0.7 ns. This value is slightly higher than that expected for a 10.6 kDa (taking into account ^15^N enrichment) monomeric protein (Supplementary Figure S2A–B), as determined from a correlation of τ_c_ versus molecular weight of 20 known monomeric proteins studied by ^15^N relaxation (51), suggesting that the tumbling of the protein in solution is not isotropic. According to the Stokes–Einstein–Debye equation, the obtained τ_c_ corresponds to an average hydrodynamic radius (*r*_H_) of 2.09 ± 0.06 nm. This value was in perfect agreement with that measured by DLS analyses (2.05 ± 0.11 nm) and, especially, with that calculated by Hydropro program (45) using the 20 models of Stf76 NMR structure here determined (2.07 ± 0.03 nm, see Supplementary Figure S2B). As a result, the experimental NMR and DLS r_H_, together with that calculated from the structure, provide clear indication that the protein Stf76 is monomeric under the analyzed conditions, though exhibiting a shape not completely globular.

### Stf76 is a DNA-binding protein

**Figure 2.**
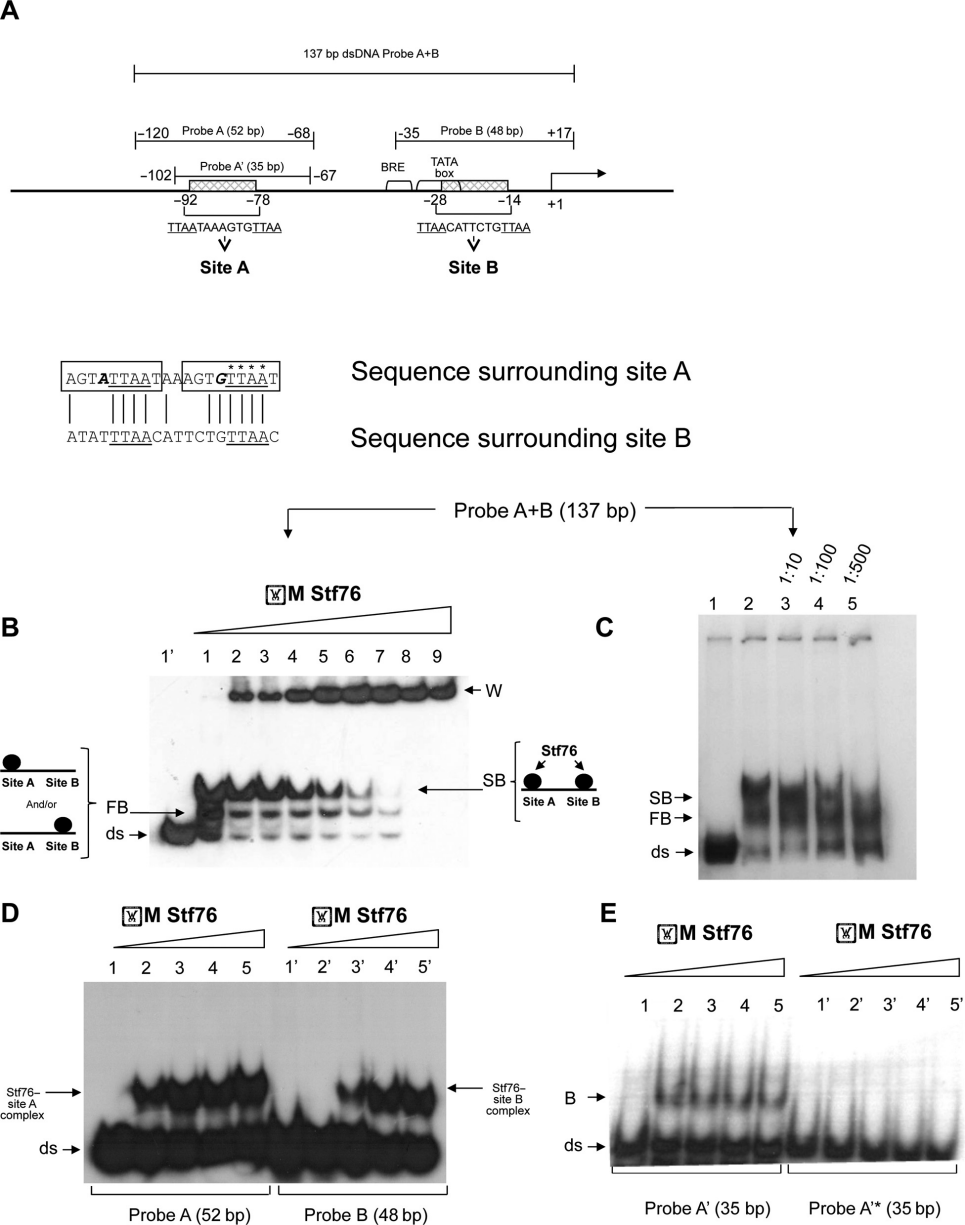
(**A**) Schematic representation of the DNA fragments used as probes in EMSAs. Sequences of site A and site B extending from −78 to −92 and from −14 to −28 with respect to the transcription start site (TSS), respectively, are shown. The two direct repeats found in site A are boxed and the nucleotides mutagenized in probe A’* are marked with an asterisk. (**B–E**) EMSA analysis of Stf76-speciﬁc binding to DNA. (**B**) Stf76 binds to a 137 bp DNA fragment (probe A+B, extending from −120/+17) that contains its own promoter in the presence of a large amount (1 μg) of non-speciﬁc competitor DNA (Salmon Sperm DNA), forming two distinct DNA-Stf76 complexes with different electrophoretic mobility. W stands for bound and aggregated DNA that remained in the wells of the gel. Binding to the labeled A+B probe (10 nM) was tested over a wide range of increasing concentration of Stf76, i.e. from 3.2, 6.4, 9.6, 12.8, 16, 19.2, 22.4, 25.8, 32.4 μM (lanes 1–9) until a complete titration of the free probe was observed. Lane 1’ contains the free probe. (**C**) Lanes 3–5, labeled A+B probe incubated with puriﬁed Stf76 (3.2 μM) in the presence of increasing amounts of a speciﬁc cold competitor DNA. The ratio between labeled/unlabeled probe is shown on the top. Lane 1 and lane 2 contain the free probe and the EMSA mixture without the cold probe, respectively. A comprehensive scheme of the interaction occurring at sites A and B (slower migrating band—SB) and at either one of the two sites (faster migrating band—FB) is shown. (**D**) Comparison between binding efficiency of Stf76 towards site A and site B. Increasing amounts of Stf76 (12.8, 25.8, 40, 51.6 μM) were incubated with 5nM of labeled probe A (lanes 2–5) and probe B (lanes 2’–5’). Lanes 1 and 1’ contain the free probe. (**E**) Identification of Stf76 binding determinants. Mutation of probe A’* at the four nucleotides indicated in Figure 2A abrogates Stf76 binding. Increasing amounts of Stf76 (12.8, 25.8, 40, 51.6 μM, lanes 2–5 and 2'–5') were incubated with 5 nM of labeled probe A' (lanes 1–5) or probe A'* (1'–5'). B is bound DNA.

As already reported, the pRN1 protein ORF80 and Stf76 share 76% of sequence identity, thus they are very likely homologs (19). The DNA-binding region of ORF80 has been extensively characterized by footprinting and EMSA analyses and identified in the operator sequences (**TTAA**N_7_**TTAA**) occurring twice in the region upstream of its own gene at a relative distance of 60 bases from each other. Both binding sites contain two palindromic TTAA motifs whose centers are separated by 11 bp, i.e. about one helix turn (19). In order to confirm that the homology between ORF80 and Stf76 corresponded also to a functional similarity, we analyzed the DNA-binding activity of recombinant Stf76 by EMSA experiments using DNA fragments issued from the region upstream of the *Stf76* gene. The putative binding sites for Stf76 were predicted to be two DNA regions identically spaced and structured to those of ORF80 (site A and site B, Figure 2A) and sharing 93–96% identity with them (Supplementary Figure S3). When a large 137 bp DNA region (from −120 to +17), including both putative binding sites (probe A+B, Figure 2A), was used as substrate in EMSA assay, Stf76 formed two concentration-dependent migrating complexes, indicating that the interaction presumably occurred at the two distinct previously indicated binding sites (Figure 2B). A third band, unable to enter into the gel (Figure 2B, W), probably resulted from non-specific Stf76–DNA interactions or from the formation of aggregates. The faster migrating complex (FB, Figure 2B) likely consists of a heterogeneous mixture of complexes exhibiting identical migration velocities in EMSA, but having Stf76 bound at one of the two target sites only, whereas the slower migrating complex (SB, Figure 2B) could correspond to Stf76 bound to two sites. The appearance of the two migrating complexes occurs almost simultaneously at Stf76 concentration of ≥2 μM (Figure 2B and Supplementary Figure S4) whereas at lower values the faster migrating band appears more defined compared to the slower one (Supplementary Figure S4). Differently from EMSA experiments performed with ORF80, we only observed two distinct bands and no intermediate migrating complexes have ever been detected (19). The calculated Hill coefficient from this titration is of 4.5, indicating the existence of a positive cooperativity in the Stf76 binding to the two sites. This value is in perfect agreement with that obtained for ORF80 and provides the minimum number of Stf76 molecules that bind cooperatively to the two sites (19). Binding of Stf76 to the analyzed region turned to be specific. Indeed, Stf76 was completely unable to bind to the promoter of the *f55* gene (52) used as negative control for EMSA experiments (Supplementary Figure S5). Furthermore, interaction with the 137-bp-long fragment was only slightly affected by the presence of increasing amounts of non-specific competitor DNA (Salmon Sperm DNA) at up to a 2000-fold excess (Supplementary Figure S6A). Conversely, the two complexes were displaced by increasing amount of cold specific DNA (Figure 2C). In detail, the slower migrating signal disappears at 500 molar excess, while the faster one still persists and decreases significantly at 1000 molar excess (Supplementary Figure S6B). Altogether these results demonstrate that Stf76 binds specifically to two distinct sites located in its own promoter.

Subsequently, in order to identify unambiguously the two interacting sites, we performed binding assays using double-stranded probes shortened step-wise and containing either one of the two putative binding sites. First, we tested ∼ 50 bp long probes (probe A and probe B, Figure 2A), and in both cases, only one retarded band was observed, indicating that each of the probes used did contain only one of the two putative binding sites (Figure 2D). By comparing the sequences of probe A and probe B (not shown), it was observed that only the regions including sites A and B shared a significant degree of similarity, thus supplying further indication that the interaction of Stf76 might occur at the two putative binding sites. Nevertheless, Stf76 showed differential affinity towards the two sites since a Stf76 /probe A complex is clearly evident at Stf76 concentration of ≥3 μM (Supplementary Figure S6C), whereas that constituted by Stf76 and probe B is detectable at a concentration ≥25.8 μM (Figure 2D). Furthermore, by quantification of the relative signals, Stf76 bound more efficiently (up to 5-fold) to site A than site B (Figure 2D) in the concentration range of 12.9–25.8 μM (Figure 2D, lanes 2–3 versus 2'–3’). By analyzing the sequences of site A and site B (Figure 2A), an almost perfect direct repeat (agta**ttaa**taaagtg**ttaa**t) was only detected in site A, thus providing a molecular basis for the higher affinity of Stf76 toward it. Moreover, since the slower band corresponding to the Stf76 bound either to site A or site B has already been observed at Stf76 concentration of 3 μM, the binding of Stf76 to site B seems to be positively influenced by the co-presence of site A on the same DNA fragment (Figure 2B and D).

To dissect the molecular basis of the interaction between Stf76 and its DNA sequence, we focused on the most affine site A. We defined the minimal size of the binding site that allowed specific binding. A distinct protein–DNA complex was detected only for the 35 bp probe A’ (Figure 2A and E), whereas with smaller probes (25 bp and 15 bp, not shown) only aspecific aggregates were observed. Therefore, all the subsequent biochemical and structural analyses (see below) were performed with the 35 bp probe A’, which retains the ability of binding Sft76 specifically.

In order to analyze the contribution of one of the two TTAA subsites of site A (**TTAA**AAAGTG**TTAA**), the second TTAA repeat was simultaneously mutagenized in four positions (probe A’*, Figure 2A). As clearly shown in Figure 2E, the binding of Stf76 was completely abolished when the mutagenized probe was used, thus confirming that at least one of the four nucleotides in the TTAA repeat is necessary for the binding to the DNA and that Stf76 interacting core sequences are indeed those initially hypothesized from comparison with the homologue ORF80 protein. Moreover, Stf76 is unable to form a stable complex with only one TTAA motif within each recognition site (Figure 2E).

### Stf76 belongs to the ‘winged helix’ DNA-binding domain superfamily

Uniformly, ^15^N- and ^15^N/^13^C-labeled Stf76 samples were used for NMR structural studies. [^15^N, ^1^H] HSQC spectra showed good cross-peak dispersion in both nitrogen and proton dimension, indicative that Stf76 adopted a well-defined structure in aqueous solution.

**Figure 3.**
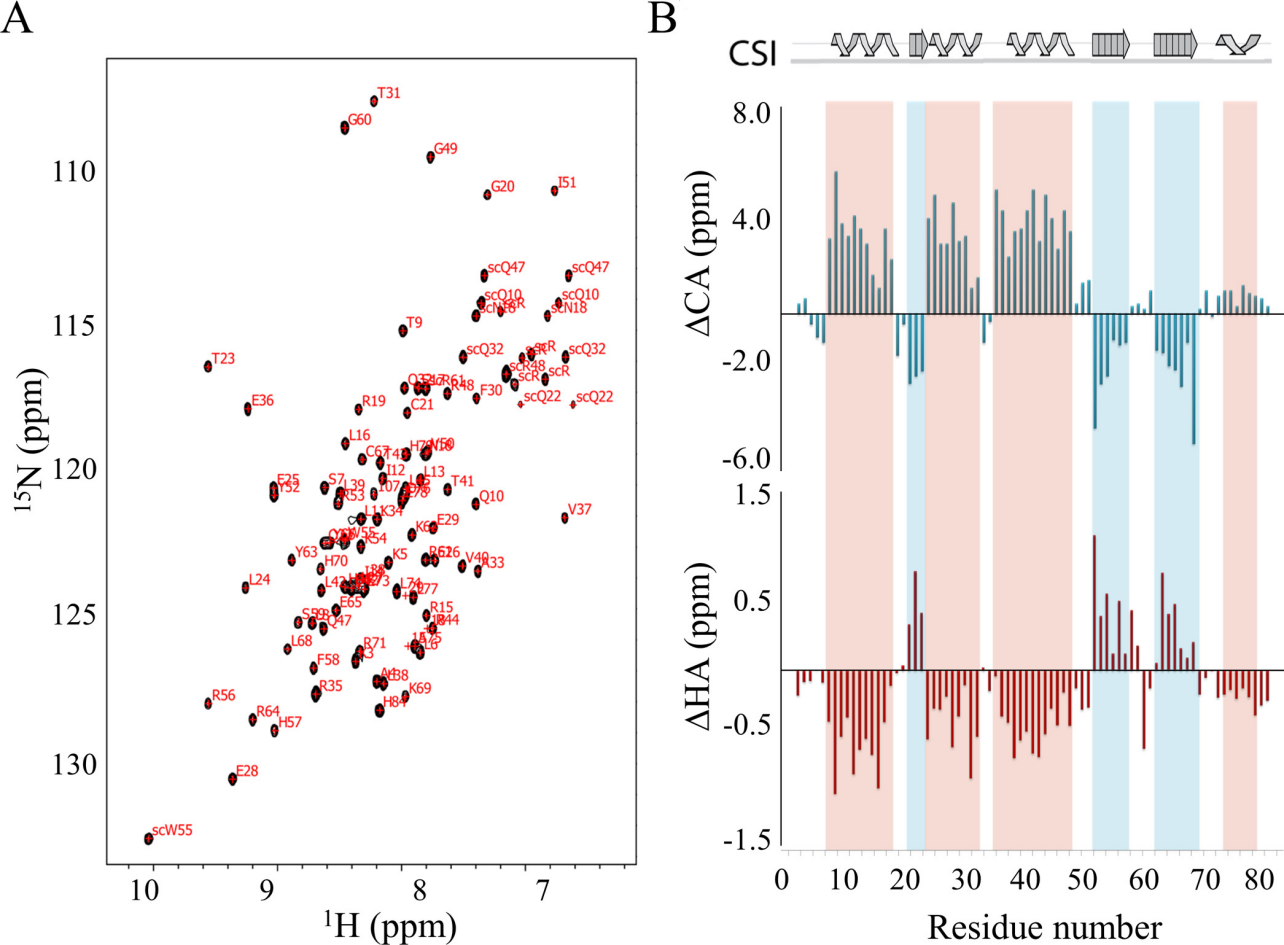
(**A**) 2D [^1^H, ^15^N] HSQC of Stf76 at 25°C and pH 5.5. Cross-peaks of the backbone amides are labeled by the one-letter amino acid code and residue numbers. Cross-peaks of side chain HNs are labeled with side chain (SC). (**B**) Chemical shift deviation from random coil values of Cα and Hα backbone atoms, obtained from CSI analysis, plotted as a function of residue number. Secondary structure elements derived from CSI are indicated above the plots. Red and blue shaded boxes indicate helical and β-strand regions, respectively.

Nearly complete backbone assignment (H^N^, N, Cα and Hα) was carried out by using data from the HNCA, HN(CO)CA, ^15^N-edited NOESY and ^15^N-edited TOCSY spectra. The few missing backbone assignments correspond to the residues of the N- and C-terminal ends (residues 1–2, 80–83). Side chain assignment could not be attained due to the relatively low protein concentration used to obtain Stf76 in the monomeric form. However, the side chain HN groups of Trp and Arg, as well as side chain NH_2_ resonances of Gln and Asn, were detected and assigned, allowing a complete assignment of the ^15^N-HSQC-detected cross-peaks (Figure 3A).

**Figure 4.**
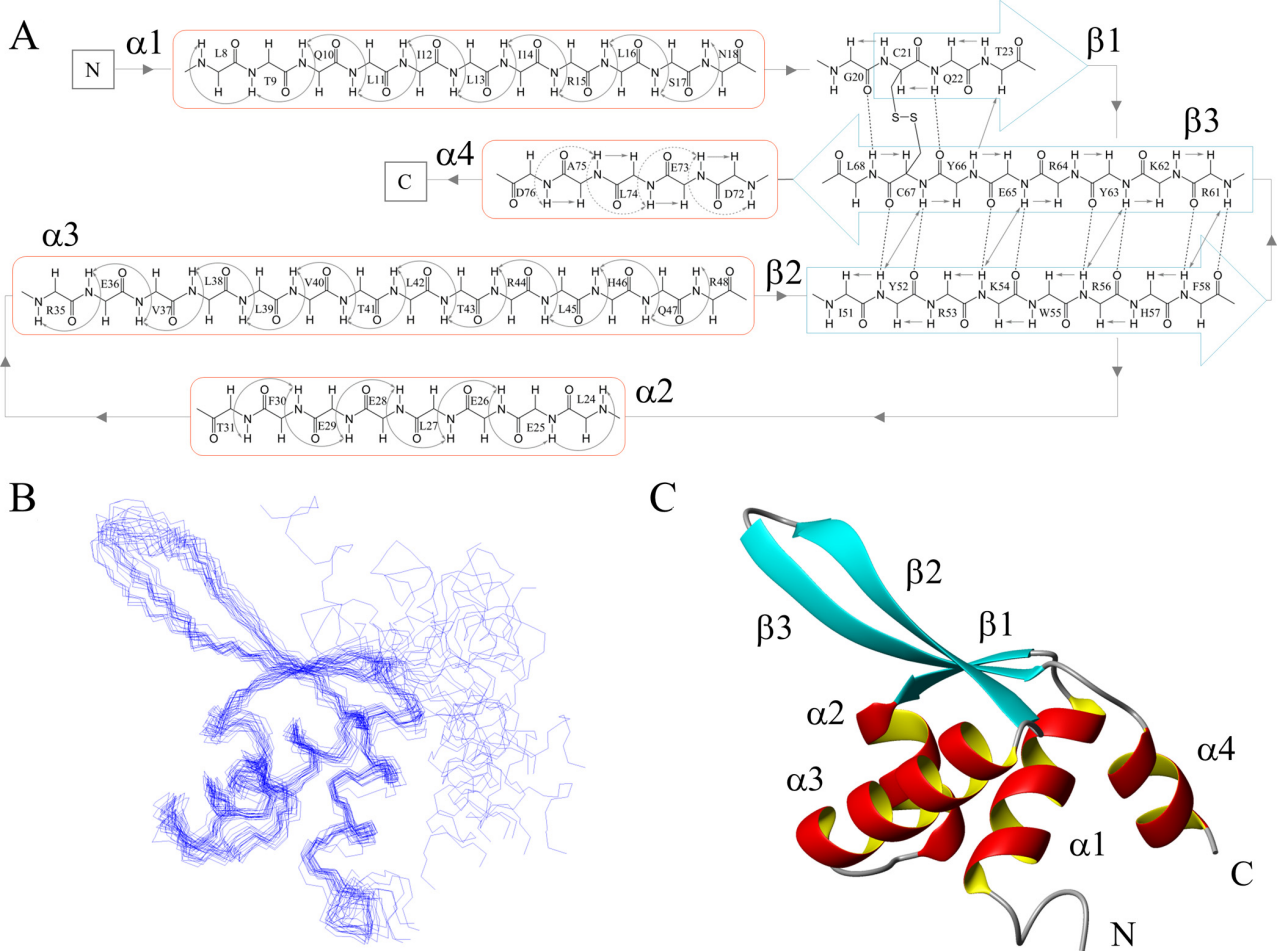
Topology and NMR solution structure of Stf76. (**A**) NOE network of four α-helices and the three β-strands of Stf76 as observed from the 3D NOESY-edited spectrum. NOEs are indicated by continuous arrows. In the α4 helix, weaker NOEs are indicated by dotted arrows. Hydrogen bonds are indicated by dotted lines. (**B**) Superimposition over the backbone atoms of residues 8–68 of the best 20 NMR structures obtained from CS-Rosetta (22,23). The disordered residues 1–2 and the C-terminal His-tag were omitted in the calculation (**C**) Ribbon diagram of the most representative structure of Stf76.

Secondary structure elements and their succession along the primary sequence were initially identified by CSI analysis (35), on the basis of the differences in experimental Cα and Hα chemical shift with respect to random coil values (ΔCα and ΔHα) (Figure 3B). This analysis revealed that Stf76 contained four α-helices and three β-strands, arranged in a αβααββα topology. These secondary structures were also confirmed by specific cross-peak patterns observed in the 3D ^15^N-NOESY-HSQC (Figure 4A).

Due to the low concentration used, high-resolution structure determination with conventional NMR-derived distance restraints and *J*-couplings could not be derived. Therefore, the 3D structure of Stf76 was determined, as reported in Materials and Methods, from NMR chemical shifts by using the CS-Rosetta software (22,23).

The final ensemble of Stf76 containing the best 20 conformers (Figure 4B) is well defined in the region encompassing residues 8–68, as indicated by the root-mean-square deviations (rmsd) with respect to the mean coordinate position of the backbone and of the heavy atoms of 0.74 Å and 1.25 Å, respectively. Residues 1–7 are mostly disordered, whereas residues 72–76 showed a propensity for helix conformation, according to their deviation from random coil values (Figure 3B). PROCHECK statistics indicate that the structures have a good quality. Indeed, all backbone ϕ/ψ pairs lie mainly within the most favored and additional allowed regions of the Ramachandran plot (53), and G-factors for dihedral angle and main-chain bond lengths are positive (Table 1).

**Table 1.** NMR structural statistics

**Structure precision**	
rmsd from mean structure (residues 5–69) (Å)	
All backbone atoms	0.99
All heavy atoms	1.69
**Structure quality**	
MOLPROBITY	
Clash score	0.89 ± 0.95
Poor rotamers (%)	0.05 ± 0.22
MolProbity score	0.71 ± 0.27
Residues with bad bonds (%)	0 ± 0
Residues with bad angles (%)	0 ± 0
Cβ deviations >0.25 Å	0 ± 0
PROCHECK	
G-factors phi-psi/all dihedral angles	0.32/0.49
Ramachandran plot statistics (%)	
Most favored regions	97.6
Additional allowed regions	2.4
Generously allowed regions	0
Disallowed regions	0

The NMR structure of Stf76 reveals a winged helix–turn–helix fold (wHTH). This fold typically contains a right-handed three helix bundle that defines the HTH motif, flanked by a two- or three-stranded antiparallel β-sheet that constitutes the wing. In the case of Stf76 (Figure 4C), the polypeptide runs from the N-terminus, through helix α1 (Leu8-Asn18), strand β1 (Cys21-Thr23), helices α2 (Leu24-Thr31) and α3 (Arg35-Arg48), and then adds strands β2 (Ile51-Phe58) and β3 (Arg61-Leu68) to complete the wing. The three-stranded version of the wHTH domain of Stf76 is often encountered in DNA-binding domains of some of the largest families of prokaryotic TFs, as well as of several eukaryotic DNA-binding domains (54). In Stf76, β2 and β3 strands are connected by a tight 1→4 turn of type I (Phe58-Arg61) and form an antiparallel β-hairpin. Three double inter-main-chain H-bonds between Tyr52-Cys67, Lys54-Glu65, Arg56-Tyr63 and Phe58-Tyr61 stabilize this hairpin (Figure 4A). The loop between helix α1 and helix α2 assumes an extended conformation and is incorporated as the third strand in the sheet via a double H-bond between backbone atoms of Tyr66 and Gln22. This arrangement brings the Cys21 close to the Cys67, confirming the presence of the disulphide bond between the two cysteines, as identified by LC-MS analysis (see above). This intramolecular disulphide bond may contribute to high Stf76 thermal stability as observed by CD studies.

^15^N longitudinal (R_1_) and transverse (R_2_) relaxation constant rates were measured to support the structural data (Supplementary Figure S1). R_1_ and R_2_ parameters are generally constant along the whole wHTH domain as expected for a rigid structure, whereas they are outside the mean values in the N- and C-terminal regions. Therefore, these data confirm that Stf76 adopts in solution a quite rigid domain in the region 8–68 with a flexible tail in the N-terminal region and a relatively more rigid C-terminal part. Noteworthy, residues of β2–β3 loop (S59, G60) show higher values of both R_1_ and R_2_ compared to the mean plus one standard deviation, indicating that these regions may exhibit nanosecond motions as well as a mobility in the micro- to millisecond timescale. Higher than average R_2_ values were also shown by residues of the α1 helix (L11), α1–α2 loop (G20), α3–β2 loop (K54), β2 sheet (H57) and β3 sheet (Y66), which may be a result of the presence of a low-frequency conformational or chemical exchange contribution.

### Structural comparison of Stf76 wHTH with related structures

A BLASTp search using the amino acid sequence confirmed that Stf76 is closely related to plasmid regulatory proteins from *Sulfolobus* (19). However, none of these proteins has been structurally characterized so far and DNA-binding activity studies are available only for Orf80.

**Figure 5.**
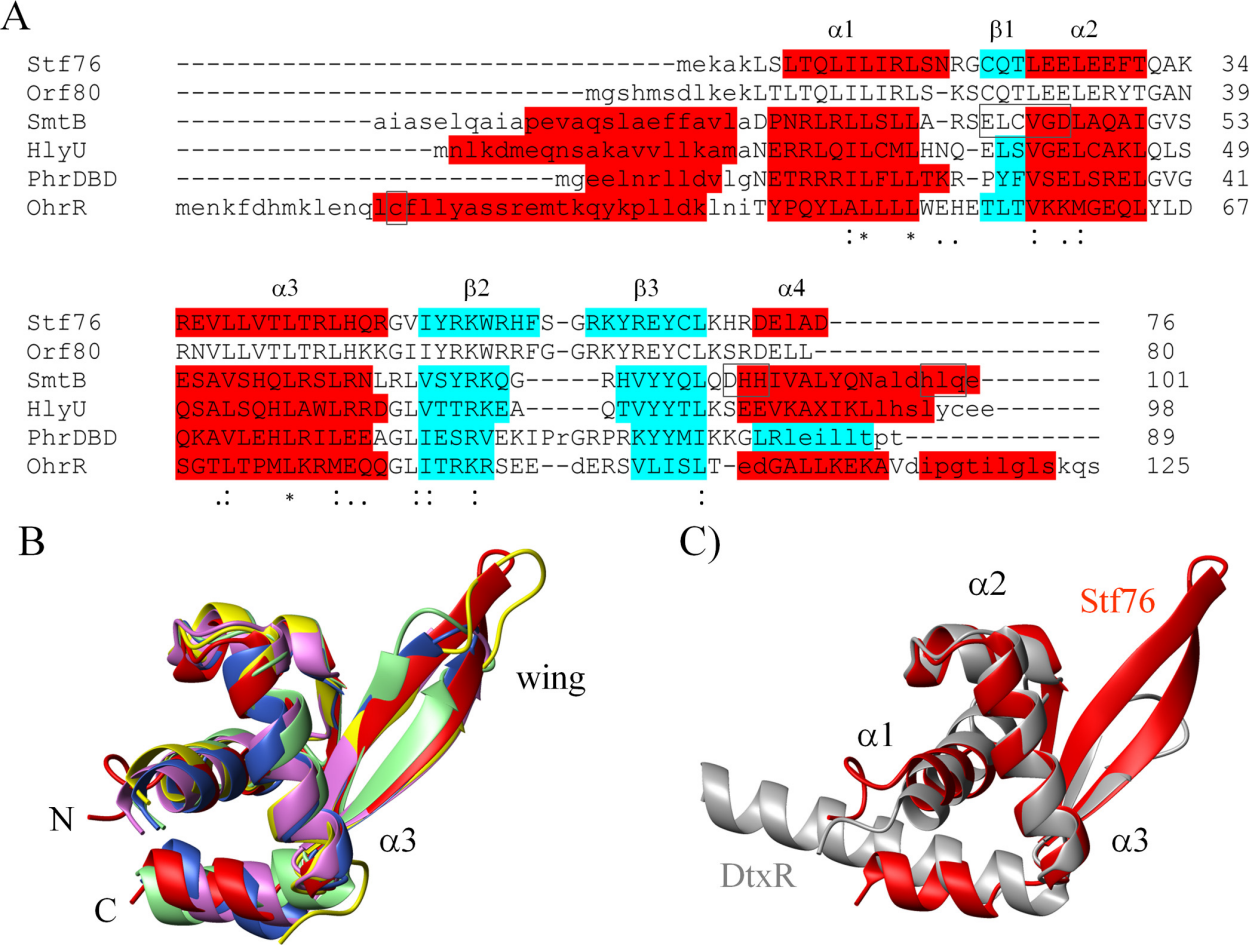
Sequence and structure comparison of Stf76 with related proteins. (**A**) Sequence alignment of Stf76 with its homolog ORF80 and structure-based sequence alignment of Stf76 with its closely structurally homologues SmtB, HlyU, Phr-DBD and OhrR. α-helices are shown in red, β-strands in cyan. Identical residues, strongly similar residues and somewhat similar residues are marked by an asterisk, a colon and a dot, respectively. Dissimilar residues are not marked. Structurally equivalent residues are in uppercase, structurally non-equivalent residues are in lowercase. The metal-binding sites in SmtB and OhrR C15, oxidized by organic hydroperoxides, are boxed. (**B**) Superimposed ribbon structures of the winged helix DNA-binding domains of Stf76 (red), SmtB (violet) (PDB ID 1smt chain B), HlyU (blue navy) (PDB ID 3jth chain B), Phr (yellow) (PDB ID 2p4w chain B) and Ohr (green) (PDB ID 1z9c chain D). The superposition was based on the backbone atoms of the wHTH domain excluding the α1α2 loop region and the wing loop, which show the most structural differences. (**C**) Superimposed ribbon structures over the three α-helices of the winged helix DNA-binding domains of Stf76 (red) and diphtheria toxin repressor DtxR (gray) (PDB ID 2dtr). (B) and (C) were prepared using the MolMol software (37).

A search for structural homologues using DALI server (40) provided both characterized and putative winged HTH transcriptional regulators, although the levels of amino acid sequence identity were fairly low (lower than 30%). In particular, the Stf76 structure is closely related to those of SmtB/ArsR family members, including the cyanobacterial metallothionein repressor SmtB from *Synechococcus* (55), the transcriptional regulator Hlyu from *Vibrio vulnificus* (56) and the archaeal heat shock regulator Phr from *Pyrococcus furiosus* (14), with rmsd over the wHTH Cα atoms of 1.6, 1.8 and 1.9 Å, respectively. Stf76 structure is also closely related to the MarR/SlyA family members, including the organic hydroperoxide resistance regulator OhrR protein (57) from *Bacillus subtilis* in a homodimeric complex with the *ohrA* promoter (rmsd = 2.0 Å) (Figure 5).

A structure-based sequence alignment of Stf76 with these related proteins showed that the residues strictly conserved in all the sequences belong to a hydrophobic core that stabilize the fold and that are responsible for the shared structural features (Figure 5A). High degree of 3D similarity is observed particularly for elements of the HTH motif, with the main differences in the length of the wing region (Figure 5B), which is known to play, together with the so-called recognition helix (α3 in Stf76), a crucial role in the contact with double-stranded DNA. According to the spatial arrangements of the helix triplets α1, α2 and α3, wHTH proteins have been classified into several structural families (58). Similarly to SmtB, Stf76 is most closely related to the toxin repressor family, characterized by having HTH angles of approximately 50° and sharing almost identical three-residue turns between α2 and α3 helices. Stf76 structure exhibits rmsd over the three helices of 1.2 Å with respect to diphtheria toxin repressor DtxR (59) (Figure 5C). Also in this case the most differences are located in the wing region.

Furthermore, compared to SmtB/ArsR and MarR/SlyA families, significant differences are observed in the flanking regions of the winged HTH domain; in particular, the N- and C-terminal helical extensions that form the dimer interface in members both of two families are absent in Stf76. This observation provides the rationale of the monomeric state of unbound Stf76 and suggests also a possible different DNA-binding mechanism with respect to these protein families.

### Biochemical characterization of Stf76 DNA-binding properties

To structurally characterize the DNA-binding properties of Stf76 to site A, different analyses were performed using 35 bp-site A (see above).

**Figure 6.**
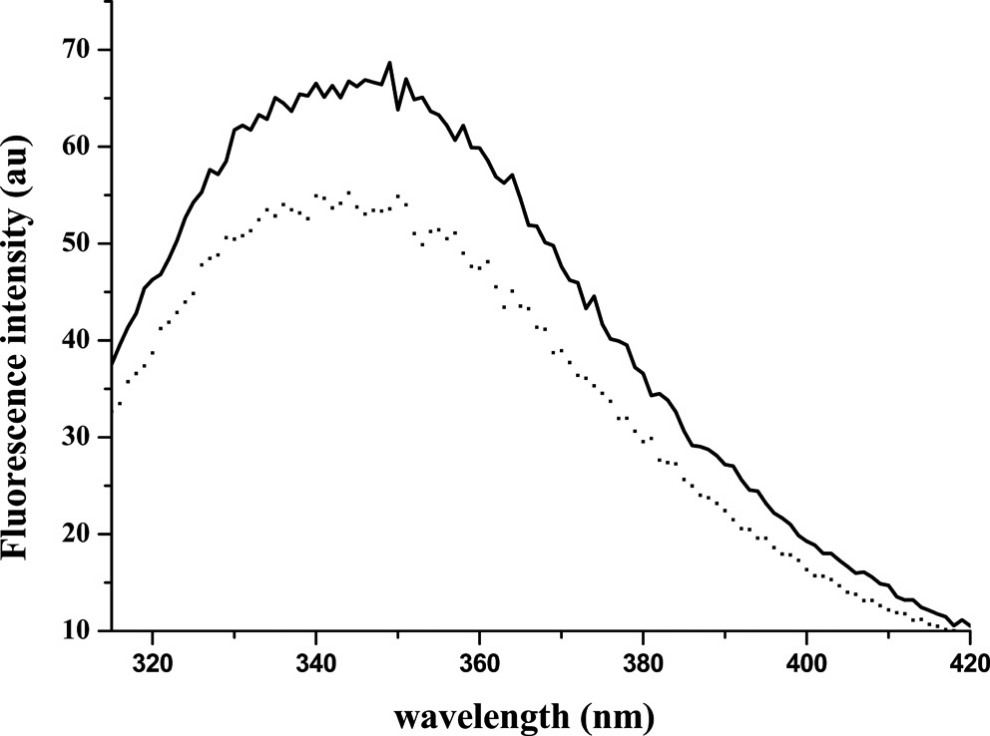
Emission spectra of the single tryptophan 55 of Stf76. Fluorescence spectra of Stf76 10 μM in the absence (continuous line) and in the presence of 5 μM of 35 bp-site A (dotted line).

The CD spectra of the protein–DNA complexes showed an increase in secondary structure elements leading to a structural reorganization of Stf76, as shown by a deeper minimum at 208 nm (Figure 1B, dashed line). The change in ellipticity increased with the amount of DNA added up to a molar ratio 2:1 (protein:DNA). Similarly, the fluorescence spectra of Stf76 registered in the presence of increasing concentration of DNA showed a decrease of emission at 355 nm until to a molar ratio 2:1 (protein:DNA) together with a slight blue-shift (Figure 6).

Moreover, the melting curve of double-stranded oligonucleotides was monitored spectrophotometrically at 260 nm in the absence and presence of stoichiometric amounts of Stf76 (2:1). The DNA was stabilized by 10°C upon formation of the complex with Stf76, indicating that melting of the protein-bound double-stranded oligonucleotides requires more energy (data not shown).

When chemical cross-linking was performed in the presence of DNA, two predominant bands with an apparent molecular mass of 10 kDa (monomer) and 20 kDa (dimer) were produced and at the same time the oligomeric forms, observed in the absence of DNA, disappeared (Figure 1 A, lanes 6 and 7) suggesting the formation of a stable dimer only in the presence of DNA, indeed. 2:1 protein:DNA ratio is a recurring value in different techniques utilized.

Protein and annealed 35 bp oligonucleotide were mixed in a molar ratio 2:1 (protein:DNA) (Figure 1C, green) and subjected to light scattering experiments. A single peak, with a molecular weight corresponding to a 2:1 complex, was obtained with no indication of excess of either protein or DNA. Interestingly in the molecular ratio 1:1 (protein:DNA) (Figure 1C, purple), a single peak with a molecular weight corresponding to a 1:1 complex was observed indicating that the protein is also able to bind DNA as monomer. In addition, the 1:1 complex eluted at a volume similar to free DNA (Figure 1C, blue), while remarkably 2:1 complex eluted at a larger volume. The behavior of the observed complexes could be justified from the formation of more compact species (Figure 1C).

**Figure 7.**
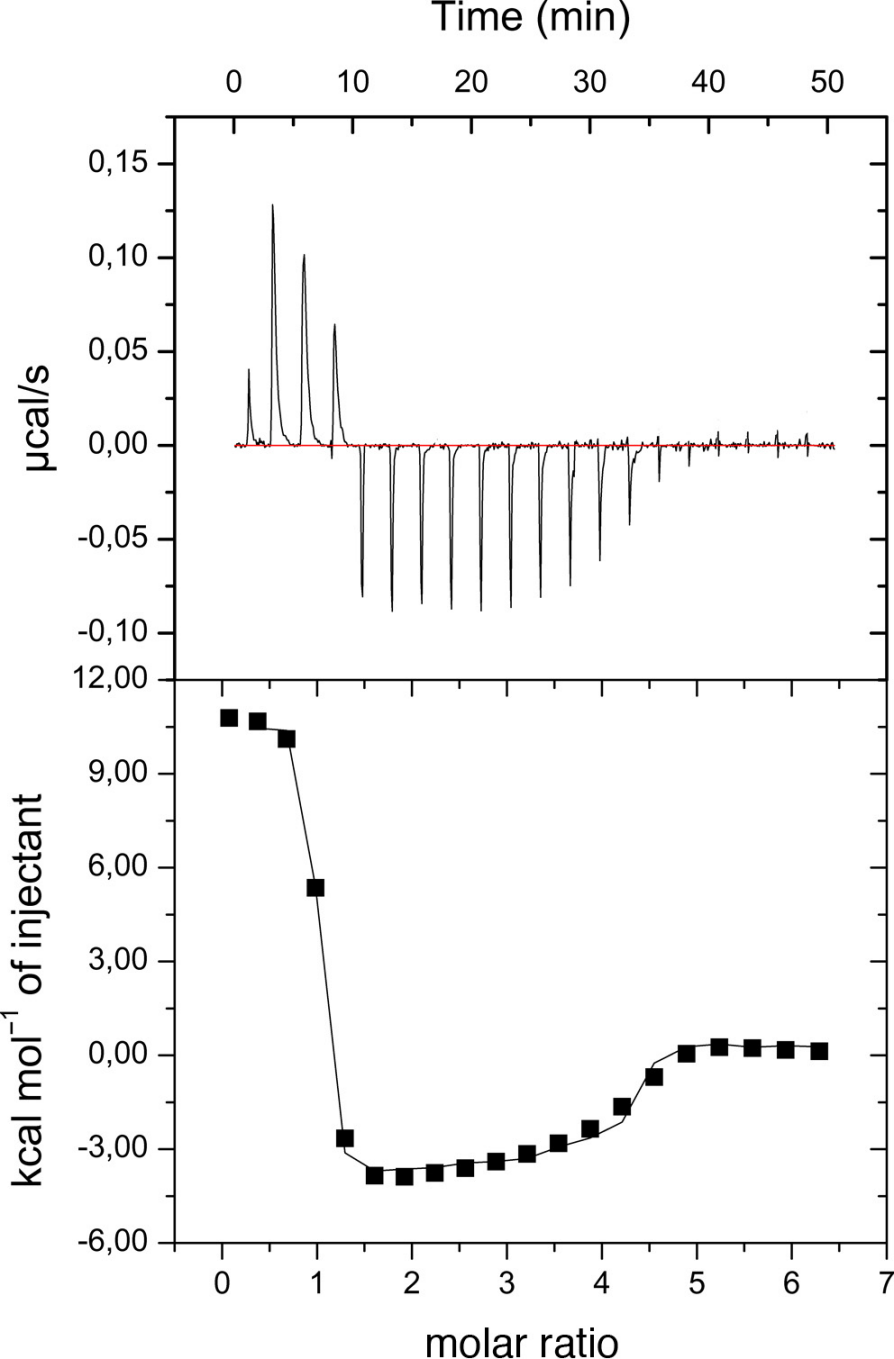
ITC data showing titration of 35 bp-site A (10 μM) with Stf76 (100 μM). The top and bottom sections report the raw and integrated data, respectively.

To assess the affinity of Stf76 for dsDNA, the energetics of the DNA-binding interaction between Stf76 (titrand) and the selected 35 bp-site A (titrant) were determined by ITC. ITC experiment on the binding of Stf76 to 35 bp-site A showed a pattern of binding isotherm that is inconsistent with a simple 1:1 complex (Figure 7). The calorimetric isotherm is biphasic, showing endothermic heat pulses for initial injections, becoming exothermic with later additions. The ITC data showed two binding events that could be well fitted to a Sequential Binding site Model. The first binding event, with significantly higher affinity (*K*_1_ = 40 nM), reached a plateau at about 2-fold ratio protein–DNA, while the second binding event at about 5-fold exhibited a lower binding constant (*K*_2_) in the micromolar range. In addition, ITC measurements also showed that the tighter binding is endothermic and, interestingly, entropy driven with an unfavorable enthalpic contribution (ΔH: 10 Kcal/mol and ΔS: 70 cal/mol/deg (Figure 7).

### Stf76–DNA interaction by NMR CSP Analysis

**Figure 8.**
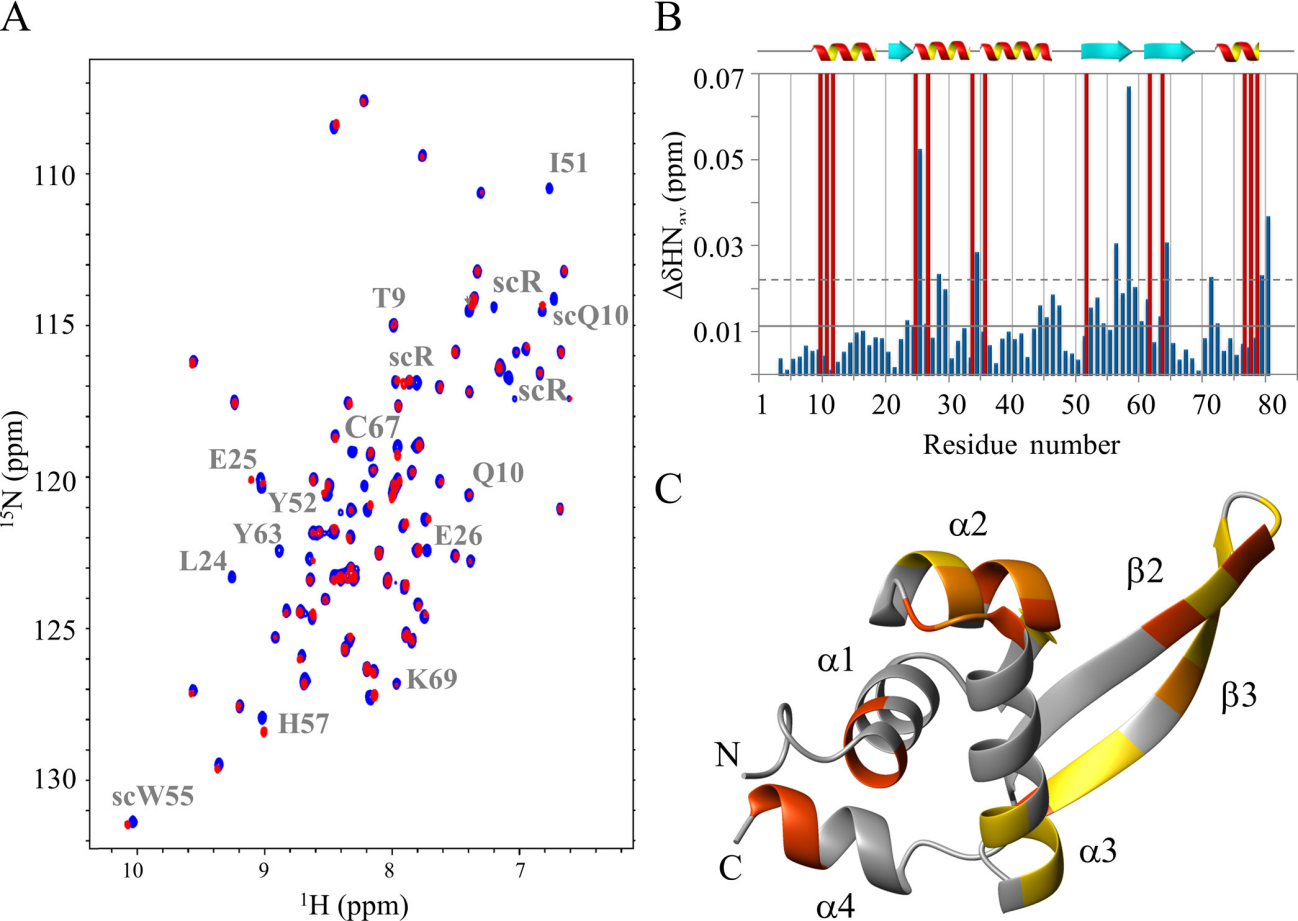
NMR CSP analysis of Stf76 upon DNA binding. (**A**) Superposition of a 2D [^1^H, ^15^N] HSQC section of Stf76 in the absence (blue) and in the presence of sub-stoichiometric amount of DNA (35 bp-site A) (red). Amide cross-peaks with significant perturbations are indicated. (**B**) Bar graphs of the average combined chemical shift differences (ΔHN_av_) as a function of residue number. The mean value is shown as a continuous line; the mean value plus one standard deviation, as a broken line. Red bars at the maximum value in the graph represent residues with the most relative intensity reduction. The secondary structure elements are also indicated. (**C**) CSP mapping onto the representative conformer of the NMR structure of Stf76 shown as ribbon drawing. Residues for which ΔHN_av_ > mean+SD and ΔHN_av_ > mean are shown in orange and in gold, respectively. Residues with the most relative intensity reduction are shown in orange red. (C) was prepared using the software MOLMOL (37).

To identify the DNA-binding interface of Stf76, we performed an NMR CSP analysis by acquiring 2D [^15^N, ^1^H] HSQC spectra of ^15^N-labeled Stf76 in the absence and presence of the 35 bp-site A sequence (Figure 8). Upon addition of a stoichiometric amount of DNA, corresponding to 2:1 Stf76–DNA complex of ∼35 kDa, the HSQC cross-peaks of Stf76 broadened away, consistent with the formation of a large, slow tumbling complex formed between DNA and Stf76, as also indicated in the light scattering and ITC studies. At sub-stoichiometric levels of DNA, however, differential broadening and chemical shift variations were observed, providing indications on the residues of Stf76 that are direct involved in the DNA interaction (Figure 8A). Averaged combined chemical shift difference (ΔHN_av_) and intensity changes of the amide cross-peaks upon DNA binding were plotted versus the residue number (Figure 8B) and the mapping on the structure of Stf76 of the residues showing significant changes is also reported (Figure 8C). Significant chemical shift and/or intensity changes can be observed for the residues located in the N-terminal region (Thr9, Gln10 and Leu11), in the helix α2 (Leu24, Glu25, Glu26, Glu28, Glu29), in the loop between α2 and α3 (Ala33, Lys34), in the helix α3 (Arg35, Arg44, His46, Gln47) and in the wing (Ile51, Tyr52, Arg53, Trp55, Arg56, His57, Arg58, Gly60, Lys62, Tyr63). Interestingly, all residues are mostly located on a single side of Stf76 protein, constituted by the helix α3 and the β2 and β3 strands (Figure 8C). Moreover, the distribution of the electrostatic surface potential indicates that one side of the protein has a continuous positively charged patch which includes the potential nucleic acid binding motif. On the opposite side, there are only two small patches positively charged, which do not fit with the CSPs (Supplementary Figure S7), indicating that this region is not involved in the DNA binding.

## DISCUSSION

Viruses and plasmids represent attractive models to understand complex biological mechanisms, such as transcription, the cell cycle and regulation of gene expression because of their small genomes that are easy to study. Among crenarchaeal genetic elements, the pRN1 plasmid and plasmid/virus hybrid pSSVx have been biochemically and physiologically characterized (15,18,21). An open reading frame of pRN1, ORF80, was functionally characterized and demonstrated to be a sequence-specific double-stranded DNA-binding protein, even if no structural analyses are available so far (19,20).

In this paper, a novel protein Stf76, encoded by the hybrid plasmid–virus pSSVx and homolog of ORF80, has been functionally and structurally analysed to shed light on the molecular mechanism underpinning its interaction with DNA as well as on its biological function.

Like ORF80, Stf76 binds to its own promoter specifically at a 35-nt sequence including a 15-nt motif (TTAA-N_7_-TTAA). The 15-nt sequence recurs twice in the region upstream of the *Stf76* gene, once (site B) in a partial overlapping arrangement with the B recognition element (BRE) element, thus suggesting a negative regulatory role of these cis-acting elements; in the second instance (site A), it localizes 84 nucleotides upstream of the transcription start site and therefore in a DNA region quite uncommon for a binding site of a prokaryotic transcriptional regulator. The two Stf76 binding sites are not equivalent since the recombinant protein interacts more strongly with the distal (site A) than with the proximal site (site B) (Figure 2D). The differential affinity can be explained at molecular level by the fact that while the former contains two almost perfect direct repeats (AGTATTAATAAAGTGTTAAT), site B lacks of this kind of symmetry (Figure 2A). After mutagenesis of one of the two TTAA sub-sites, the binding ability of Stf76 is completely abrogated, as shown by EMSA assay, further supporting the hypothesis that both sub-sites (5’-TTAA-3’) are required for the formation of stable complexes on the DNA. Our EMSA data indicate not only that the TTAA core of the motif is essential for the binding, but also that the flanking nucleotides might play a relevant role in the stabilization. Overall, our data were in good agreement with those observed in the atomic force microscopy (AFM) study of the ORF80 interaction with dsDNA in which two ORF80 molecules were visible to bind one TTAA-N7-TTAA motif. Single complexes, preferentially formed by interaction of ORF80 to one of the two sites, were also clearly distinguishable. Interestingly enough, the most affine ORF80 site corresponds to Stf76 site A.

The NMR structure of Stf76, here determined, indicates that Stf76 belongs to the wHTH superfamily (Figure 4), which includes the majority of *Sulfolobus* TFs. Since ORF80 and Stf76 share 76% of sequence identity, the sequence homology likely corresponds also to a structural homology (Figure 5A). ORF80 was initially predicted to bear a leucine zipper motif on the basis of the position of five completely conserved leucine (19). Subsequently, it was hypothesized to be a winged helix protein, as it exhibits a weak similarity in amino acid sequence and (predicted) secondary structure to several sequence-specific DNA-binding proteins with the wHTH fold (20). Our data confirm indirectly that also ORF80 is a novel member of the wHTH superfamily. Four of the five conserved leucine (Leu13, Leu16, Leu24 and Leu27 in Stf76) belong to the hydrophobic core that stabilize the fold, thus having a role different from that initially predicted (19).

Stf76 displays the three stranded version of the wHTH domain (54) in which the tight three-helical core is followed by a C-terminal hairpin (the wing) that forms a β-sheet with a short β-strand in the loop between α1 and α2 helices. The relaxation data confirm that region 8–68 is substantially rigid with some degree of flexibility in β2–β3 loop, whereas the N-terminus is largely flexible and the C-terminus more rigid with helical propensity (Supplementary Figure S1).

A DALI analysis showed that the Stf76 fold is structurally related to members of bacterial and archaeal SmtB/ArsR and MarR/SlyA families. SmtB/ArsR family is a class of transcription regulator involved in stress response to heavy metal ions. The structural homolog of Stf76, SmtB, is one of the best characterized members of this family (60–62). It is a homodimer that functions by binding to its operator promoter (two inverted repeats), and dissociating in the presence of Zn^2+^. The comparison of the sequence of Stf76 with SmtB shows that the metal-binding site residues of SmtB are substituted in Stf76 with residues not able to chelate any metal ions (Figure 5A), suggesting a different function for Stf76. However, also HlyU and Phr, the other two ArsR/SmtB family members closely structurally related to Stf76, do not respond to heavy metals for the absence of metal-binding sites.

MarR/SlyA family is a class of transcription regulators involved in the development of antibiotic resistance. The mechanism by which its member OhrR responds to organic hydroperoxides is based on the oxidation of the Cys15, localized in the N-terminal helical extension of the wHTH domain, to Cys-sulphenic acid. This residue, as shown in Figure 5A, is absent in Stf76, indicating also in this case an unrelated function between Stf76 and OhrR.

As expected, the wHTH domain of Stf76 is dominated by positive charges, but it shows some atypical properties with respect to canonical winged HTH proteins (Supplementary Figure S6C, D). Indeed, typical wHTH have a basic surface mainly located on the recognition helix (63). In contrast, Stf76 exhibits clustering of basic residues on the wing (R53, K54, R56, R61, K62, R64) but displays only few basic residues on the recognition helix (R34, R44, R48). Interestingly, a similar surface charge distribution is observed in the structurally related proteins (Figure 5A), suggesting a similar binding interface.

wHTH domains are often combined in the same protein with other domains or enriched with other secondary elements which contribute to protein dimerization (54). Furthermore, wHTH proteins generally bind as dimers to palindromic or direct repeat DNA sequences in which each monomer recognizes a half site. Stf76 presents further peculiar features with respect to the other members of the wHTH superfamily because it lacks the additional elements involved in the dimerization and it is a monomer in solution. Nevertheless, chemical-cross-linking, light scattering, CD, ITC and spectrofluorimetric experiments revealed that interaction between two monomers to form stable dimers occurs, but only in the presence of DNA (64). In particular, CD and spectrofluorimetric titrations indicate the formation of a 2:1 protein:DNA complex, when Stf76 interacts with the fragment 35 bp-site A.

Far-UV CD spectra of free Stf76 showed a properly folded protein with an α/β fingerprint. In the presence of DNA, CD analyses indicate that Stf76 undergoes to an increase in helical content upon binding to the target DNA. As observed by NMR CSI analyses and ^15^N relaxation studies (Figure 3B and Supplementary Figure S1), the three-helical core is well defined and more rigid, whereas the C-terminal helix, α4, shows the highest flexibility in the free protein. Therefore, upon binding to DNA, the disorder to order transition could involve the C-terminal helix of Stf76, which is indeed significantly affected by protein–DNA interaction (Figure 8).

In addition, spectrofluorimetric spectra showed that the only Trp present in the sequence (Trp55) results to be exposed in the free protein (λ_max_ emiss = 355 nm), in agreement with the NMR structure. In the presence of increasing DNA amounts Trp55 moves to a less polar environment as indicated by a slight blue-shift (λ_max_ emiss = 350 nm) with a concomitant decrease in emission due to quenching for the formation of DNA–protein complex. Accordingly, the Trp55 is located in the β2 strand of the basic C-terminal hairpin that establishes contacts with DNA as indicated by NMR data. In this context, the comparative DNA-binding model (see below) suggests that the interaction occurs with the minor groove.

Direct protein–DNA recognition interactions are usually characterized by favorable ΔH and favorable ΔS deriving from water molecules release, while those that strongly distort the DNA have net unfavorable ΔH, primarily associated with the base pair destacking (65). Our ITC data show a favorable entropic contribution and an unfavorable enthalpic change in the formation of the complex Stf76–DNA. Significant DNA distortions might be associated with the formation of stable Stf76 dimer deriving from a forced functional interaction of two monomers when bound to DNA. Accordingly light scattering data indicate the formation of a 2:1 complex, being more compact than the 1:1 complex due to a considerable DNA distortion.

Two potential pathways for DNA binding of protein dimers have been described: (i) a protein pre-exists in solution as a dimer and binds to DNA in this oligomeric form; (ii) the protein dimerizes upon sequential binding of two monomers to the DNA. Based on our results, the second pathway better describes the binding mode of Stf76 and would suggest that the shift of the monomer–dimer equilibrium dependent on the DNA interaction represents a level of regulation of Stf76 activity, as already described for eukaryotic TFs (66,67).

The close structural similarity among Stf76 and the DNA-binding domain of ArsR/SmtB and MarR/SlyA family members suggests a likewise pattern of DNA–protein interaction. Using NMR CSP studies of Stf76 in the presence of the 35 bp fragment of site A, we defined a DNA-binding surface constituted by the N-terminal portion of α1, α2 and α3 helices, the α2α3 loop and the β-hairpin of the wing region that well superimpose on the positively charged region (Supplementary Figure S4). Moreover, also some residues of the C-terminal α4 helix seem to be affected upon DNA binding, even if it seems to be not part of the surface directly involved in the DNA–protein interaction. Possibly, these residues could be involved in protein–protein interactions stabilizing the dimer, but more detailed structural data will be needed to clarify this point.

**Figure 9.**
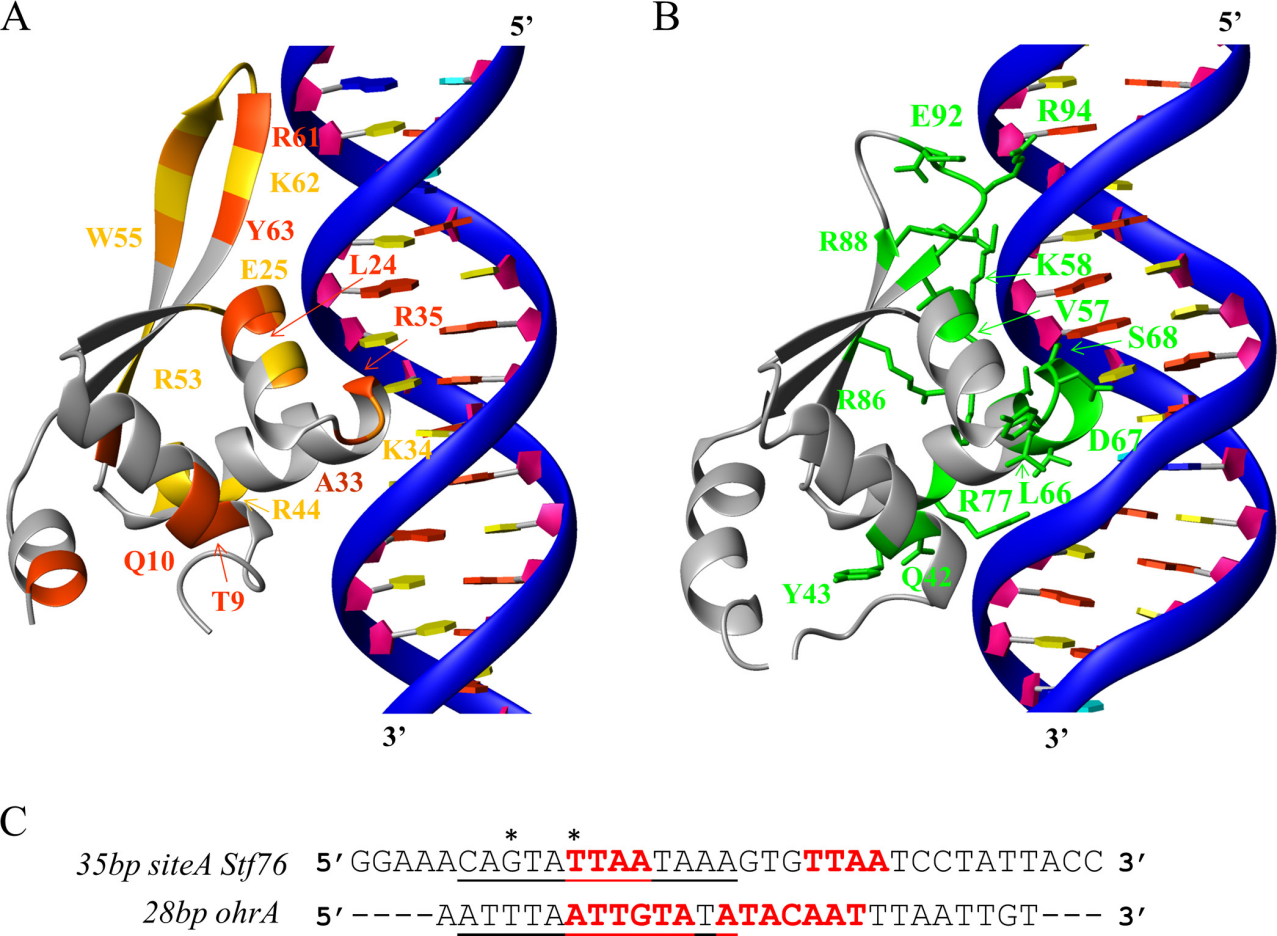
Model of the Stf76–DNA interaction. (**A**) One Stf76 monomer was positioned on a single sub-site of the 35 bp-site A on the basis of similarity with OhrR (ref). The backbone atoms of the Stf76 NMR structure were superimposed onto those of the crystal structure of the OhrR–*ohrA* DNA complex, reported in (**B**) (PDB code 1Z9C). In (A), the DNA of OhrR was replaced by a straight B form of the DNA, modeled by using 3D-DART (68). The backbone atoms of *Stf76* DNA were superimposed onto those of *ohrA* DNA, aligning the DNA sequences as reported in (**C**). The underlined bases are those represented in (A) and (B) panels. The two TTAA motifs in *Stf76* and the inverted repeats in *ohrA* are highlighted in bold red. Two bases, possibly involved in specific interactions, are indicated by an asterisk. In (A), DNA-binding elements of Stf76 are colored as in Figure 8C; in (B), those of OhrR are shown in green. Homologue residues in Stf76, that in OhrR interact with DNA, are labeled. Residues of the proteins that are not involved in DNA interaction are shown as a gray ribbon. Backbone atoms of the double-stranded DNAs are represented as ribbon in blue. A, T, G and C base rings and sugar rings are indicated as plates in orange red, yellow, green, cyan and pink, respectively.

To date, structures in complex with DNA are still unavailable for members of the ArsR/SmtB family, whereas the only complex structure available for the MarR/SlyA family is represented by the transcriptional regulator BsOhrR bound to the 28-bp duplex *ohrA* operator. Since Stf76 exhibits a high degree of structural similarity to the OhrR wHTH domain, as shown by the DALI analysis, we used the structure of the OhrR-wHTH in complex with its DNA target as a template to generate a model of a single Stf76 monomer binding to DNA. We first superimposed the backbone atoms of Stf76 wHTH domain onto those of OhrR, retaining the Stf76 binding site as the *ohrA* target DNA. Although a bent DNA model could be also plausible, in the absence of molecular experimental data we used a straight B form DNA of the 35 bp-site A. Thus, the DNA backbone atoms of *Stf76* were superimposed onto those of *ohrA* DNA, positioning the TTAA motif in the major groove, where OhrR and, hence, Stf76 establish specific contacts (Figure 9). In the modeled structure, there is a wHTH canonical mode of DNA recognition, in which the α3 helix is presented to the major groove specifically contacting DNA base pairs. The wing and the N-terminal portion also make contacts with the minor groove of the DNA. Remarkably, the hypothesized DNA-binding surface of Stf76, as determined by NMR CSP studies, is well consistent with DNA recognition mode of OhrR. In particular, DNA binding conserved residues of Stf76 on the relative position of OhrR are: Leu24 (Val57) and Glu25 (Lys58) in the α2 helix, Lys32 (Asp67) in the loop α2–α3, Arg44 (Arg77) in the recognition α3 helix, Arg53 (Arg86) in the β2 strand, Arg61 (Glu93) and Lys62 (Arg94) in the β3 strand. Most of the differences are observed in the α3 helix that could be justified by a different site-specific DNA recognition between OhrR and Stf76. In particular, Arg35 in the α3 helix of Stf76, replaced with Ser68 in OhrR, could make a specific interaction with the first thymine of the TTAA motif. In the wing of OhrR Arg94, which establishes a specific contact with the minor groove, is replaced in Stf76 with the Lys62. The latter points outside the DNA-binding site in the model and is less affected by the binding than the adjacent Arg61 and Tyr63 (see Figure 8b), letting hypothesize that one of these two residues is likely involved in DNA recognition. In particular, a specific interaction could possibly be established with the guanine, just preceding the TTAA site, analogously to what made between Arg 94 and the corresponding thymine in the OhrR–*ohrA* complex (Figure 9). Accordingly, the long wing of Stf76 contributes to an elongated shape of the protein, in line with the hydrodynamic analysis, and accounts for the requirement to contact extra nucleotides flanking the TTAA sub-site for the stabilization of specific DNA binding.

Our previous northern blot analysis indicated that the *Stf76* gene undergoes to a dual transcription regulation entailing the occurrence of a considerable activation and a complete abrogation of its own expression depending on the pSSVx life cycle and host growth (16). Indeed, mRNA level increases progressively reaching its highest intracellular concentration at the end of the exponential growth before suddenly dropping upon the onset of a strong induction of pSSVx replication. Since the binding sites of Stf76 are located within its own promoter, it is conceivable that Stf76 could play a regulative role of its own expression.

**Figure 10.**
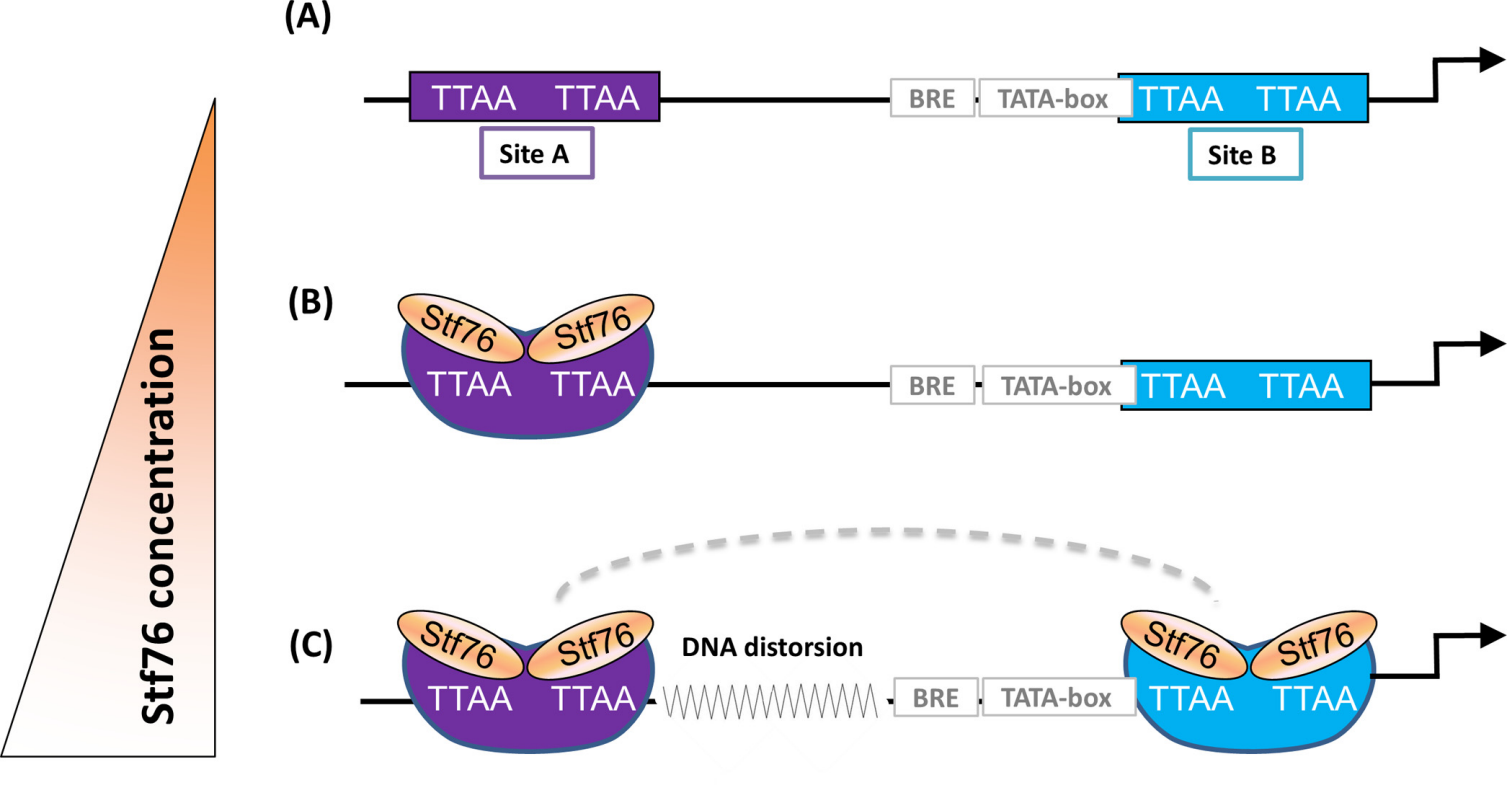
A simplified model for the Stf76 interaction to its own promoter. The two sites are saturated in a concentration-dependent manner according to the differential affinity of Sft76 toward them. (**A**) The promoter region containing the two binding sites, A and B, for Stf76. (**B**) At low concentrations, Stf76 binds only to the most affine site A. (**C**) This binding induces DNA distortion (indicated by shape modification of the binding site) that presumably extends to the adjacent regions in the promoter (as shown by wavy lines). This could lead to positive cooperative binding to site B at higher concentrations (indicated by a dotted line).

Although our data do not indicate whether Stf76 exerts its physiological function through interaction with one or both sequences (site A and site B), binding most likely starts from the most affine primary site A and subsequently extends to site B in a concentration-dependent manner. Our EMSA data indicate a positive cooperativity in the binding of Stf76 to its interacting sites. Because of the remarkable distance between site A and site B, at this stage no exact mechanism for the cooperativity observed, can be proposed. Based on the DNA distortions highlighted during ITC and light scattering experiments, one hypothesis is that Stf76 interaction to site A might induce a conformational change that favors the binding of Sft76 to site B. Since 60 bp corresponds roughly to six complete double-helix turns, the cooperativity observed might rely on the two binding sites facing the same side of the helix. A hypothetical model obtained by combining all the data here reported is presented in Figure 10. Interestingly, the putative binding sites of Stf76 homologues are well conserved both in the general structure and in the sequence. In particular, the retention of the distance between the centres of the two TTAA motifs within one binding site as well as the relative spacing of 60 bp between the two TTAAN7TTAA binding sites indicate that the regulation activity of Stf76 and of its homologues strictly depends on these specific features of the DNA-binding region.

Homologues of Stf76 are widespread among *Sulfolobus* cryptic and conjugative plasmids, suggesting a fundamental function for these TFs in the maintenance and/or in the life cycle of the plasmids in the host cells. It is tempting to speculate that Stf76 exerts its regulation activity not only by targeting its own promoter but also the regulative sequences of host genes involved in the replication and/or maintenance of the appropriate plasmids copy number (19).

Stf76 represents the first example of a viral protein belonging to the wHTH family which has been deeply characterized by means of a multidisciplinary approach including biochemical, molecular biology and NMR techniques, while other wHTH viral proteins are known only at 3D level by crystallographic techniques.

The results of this study show that Stf76 is a novel representative of the wHTH family. Compared to other members of the same family Stf76 presents several peculiar features: (i) it is a monomer in solution, (ii) it does not require additional secondary structure elements to exert its physiological function, (iii) residues that are usually responsible for effector binding in other prokaryotic wHTH TFs characterized so far are not present, (iv) the location as well as the relative distance of the two recognition motifs (sites A and B) suggests that the DNA recognition mechanism and the mode of action are unusual and potentially novel.

## ACCESSION NUMBERS

The structure and chemical shifts of Stf76 have been deposited in the PDB protein data bank (http://www.pdb.org) and the BMRB database (http://www.bmrb.wisc.edu) under the accession numbers 2MLG and 19821, respectively.

## SUPPLEMENTARY DATA

Supplementary Data are available at NAR Online.

SUPPLEMENTARY DATA
