# Supplementary material for: Structural and functional studies of Stf76 from the Sulfolobus islandicus plasmid–virus pSSVx: a novel peculiar member of the winged helix–turn–helix transcription factor family
Source: Nucleic Acids Res. 2014 Mar 25;42(9):5993–6011. doi: 10.1093/nar/gku215 (PMC4027180; doi:10.1093/nar/gku215)
Supplement: SUPPLEMENTARY DATA [file supp_42_9_5993__index.html]

Structural and functional studies of Stf76 from the Sulfolobus islandicus plasmid–virus pSSVx: a novel peculiar member of the winged helix–turn–helix transcription factor family — SUPPLEMENTARY DATA 

# Structural and functional studies of Stf76 from the *Sulfolobus islandicus* plasmid–virus pSSVx: a novel peculiar member of the winged helix–turn–helix transcription factor family

## SUPPLEMENTARY DATA

**Files in this Data Supplement:**

- SUPPLEMENTARY DATA
