# Supplementary material for: Structural and functional studies of Stf76 from the Sulfolobus islandicus plasmid–virus pSSVx: a novel peculiar member of the winged helix–turn–helix transcription factor family
Source: Nucleic Acids Res. 2014 Mar 25;42(9):5993–6011. doi: 10.1093/nar/gku215 (PMC4027180; doi:10.1093/nar/gku215)
Supplement: SUPPLEMENTARY DATA [file supp_gku215_nar-03601-h-2013-File013.doc]

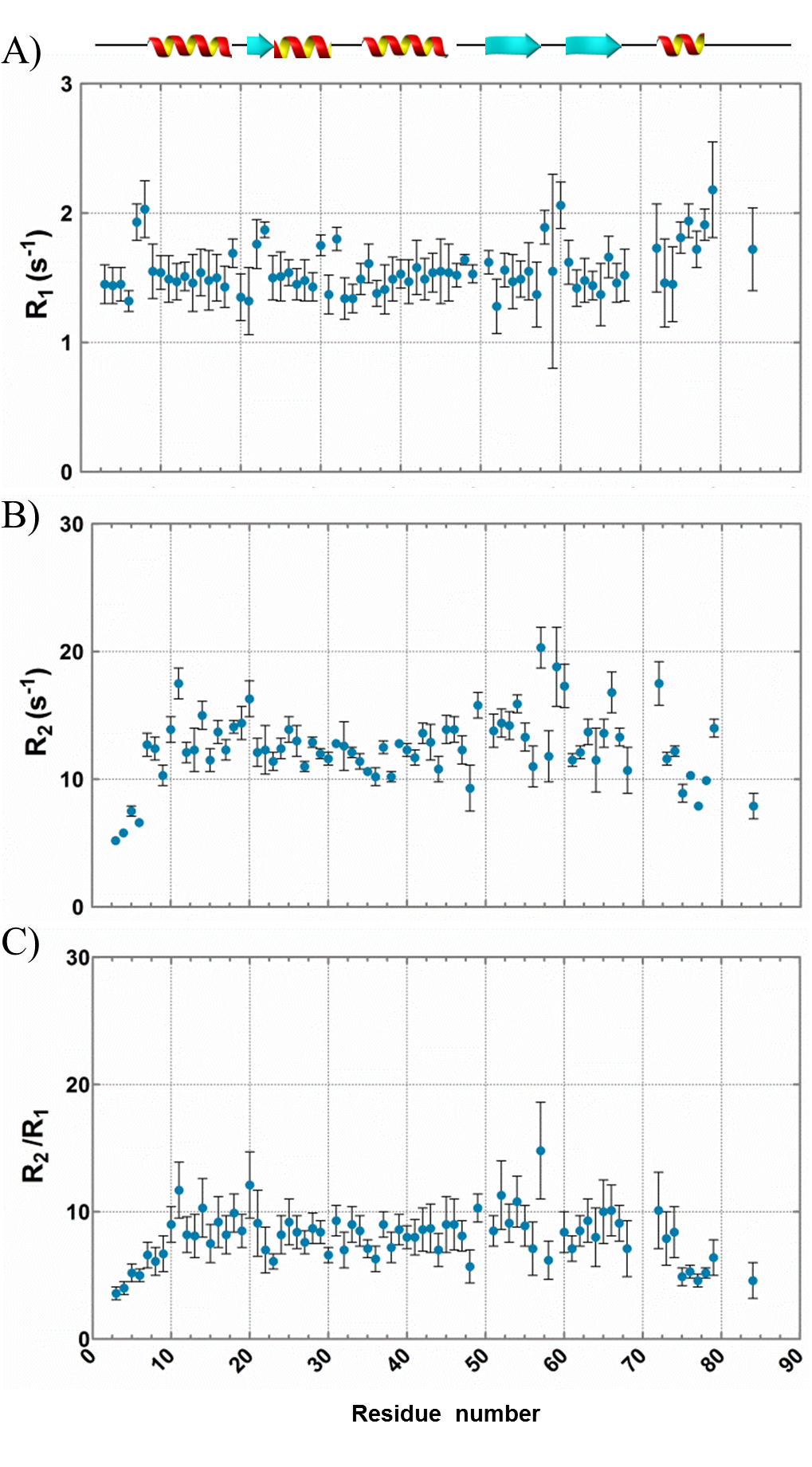


**Figure S1**. ^15^N Relaxation rates R_1_ (A) and R_2_ (B), R_2_/R_1_ ratio (C) for Stf76 at 14.1 T plotted as a function of the residue number. Error bars in plots are obtained by fitting statistical errors for R_1_ and R_2_, and by applying the error propagation law for R_2_/R_1_.


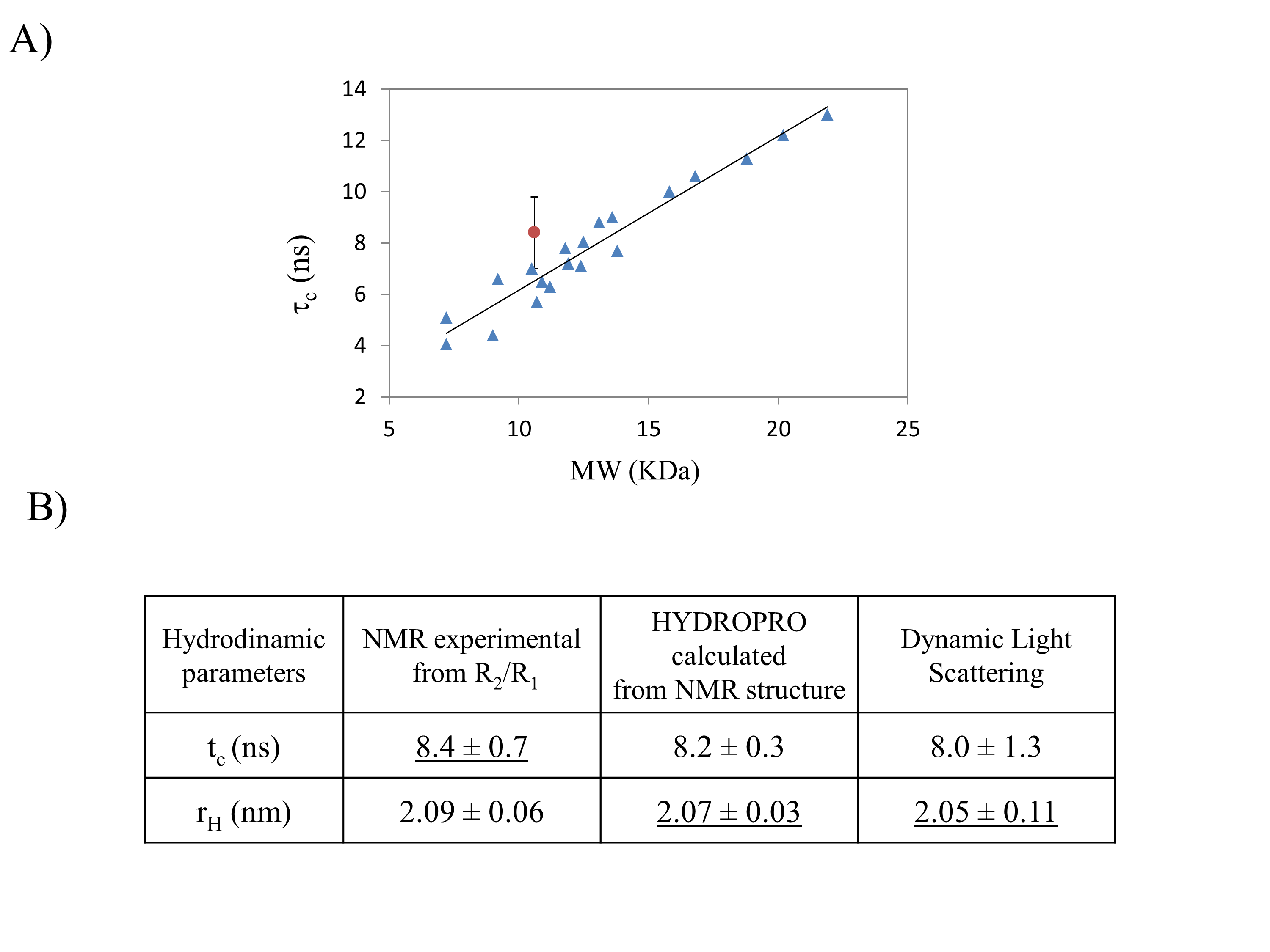


**Figure S2**. (A) Plot of the isotropic rotational correlation time, τc (ns), versus protein molecular weight (kDa) for known monomeric NESG targets of ranging size (blue triangle) (48) and for Stf76 (red circle). τ_c_ was obtained from the R_2_/R_1_ ratio measured on a 600 MHz spectrometer at 298 K for all monomeric proteins and for Stf76. (B) Comparison of hydrodynamic parameters, τ_c_ and r_H_, as determined by relaxation data, HYDROPRO software (42) based on the NMR CS-Rosetta structure and Dynamic Light Scattering measurements. The underlined values indicate those determined directly from the different approaches. For NMR and HYDROPRO methods, τ_c_ and r_H_, respectively, are mean values and corresponding errors are given from standard deviations. The other values are determined using the Debye-Stokes-Einstein equation and the errors are obtained by applying the error propagation law.


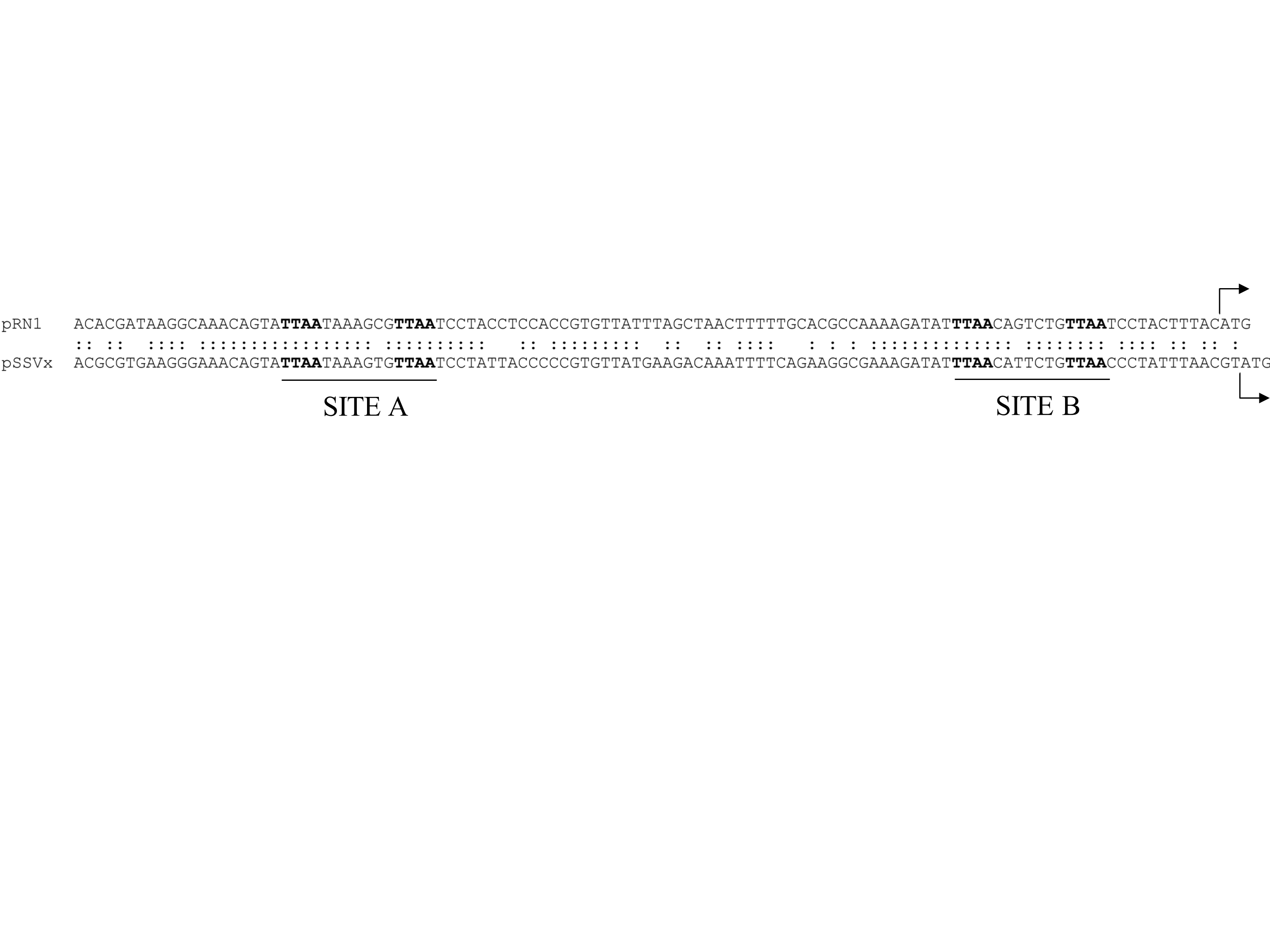


**Figure S3**. Comparison of the nucleotide sequence of the region including site A and site B of pRN1 versus that of pSSVx plasmid. The arrows indicate the transcription start sites.


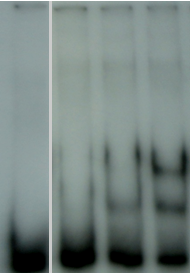


**Stf76**

**Site A**

**Site B**

**Site A**

**Site B**

**Site A**

**Site B**

**SB**

**FB**

**Or/and**

**Stf76 [μM]**

**0.5      1        2**

ds

**Figure S4**

Binding to the labelled A+B probe (10 nM) was tested over a lower range of increasing concentration of Stf76, compared to those shown in Figure 2B, i.e. from 0.5 to 2 μM. SB and FB indicate slower migrating band and faster migrating band, respectively. Ds stands for unbound DNA.


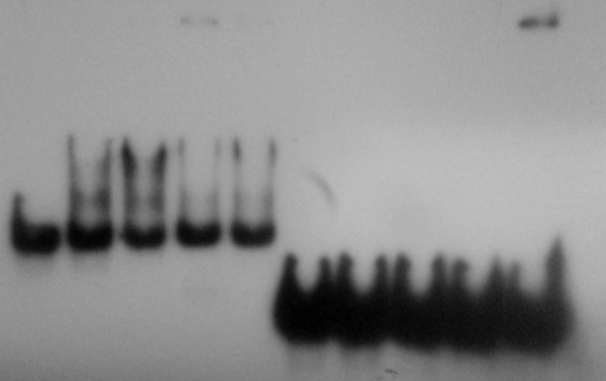


1

2

3

4

5

1’

2’

3’

4’

5’

**μM Stf76**

**μM Stf76**

**Probe A+B (137bp)**

**Probe f55 (31 bp)**

Free probe

Free probe

FB

SB

**Figure S5**

Binding activity of Stf76 toward site A+B and the aspecific probe f55. Increasing amounts of Stf76 (1, 3, 6, 12 μM) were incubated with 5nM of labelled Probe A+B (Lanes 2-5) and Probe f55 (Lanes 2’-5’). Lanes 1 and 1’ contain the free probes. FB and SB indicate Faster migrating Band and Slower migrating Band, respectively.


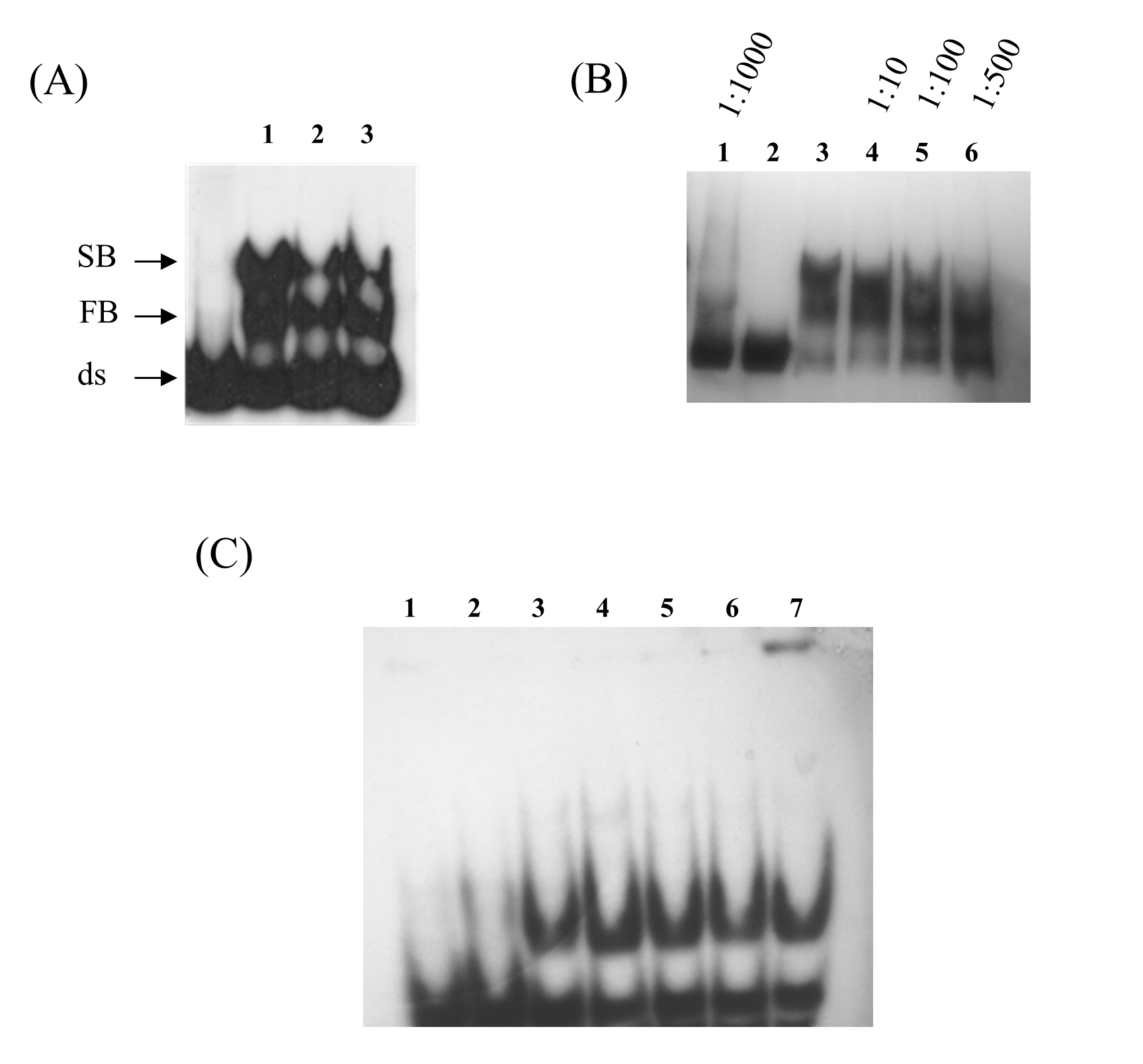


**Figure S6.** (A) EMSA of the −120/+17 region in the presence of non-specific competitor. Ds: unbound DNA probe; lane 1 , labelled probe incubated with Stf76 (3.2 *μ*M) without competitor DNA; lanes 2–3 , labelled probe incubated with Stf76 in presence of increasing amounts (1000-2000 fold, respectively) of non-specific competitor DNA (salmon sperm DNA). SB and FB indicate slower migrating band and faster migrating band, respectively. (B) EMSA experiments performed with increasing amounts of speciﬁc cold competitor DNA. Lanes 1 and 4–6: the labelled A+B probe was incubated with puriﬁed Stf76 (3.2 μM) in the presence of the ratio between labelled/unlabelled probe indicated on the top. Lanes 2 and 3 contain the free probe and Stf76 without cold competitor, respectively. (C) EMSA experiments performed with increasing amounts of Stf76 (lanes 2 to 7: 1.5, 3, 6, 9, 12, 18 μM) and 5nM of labelled probes A´. Lanes 1 contains the free probe.


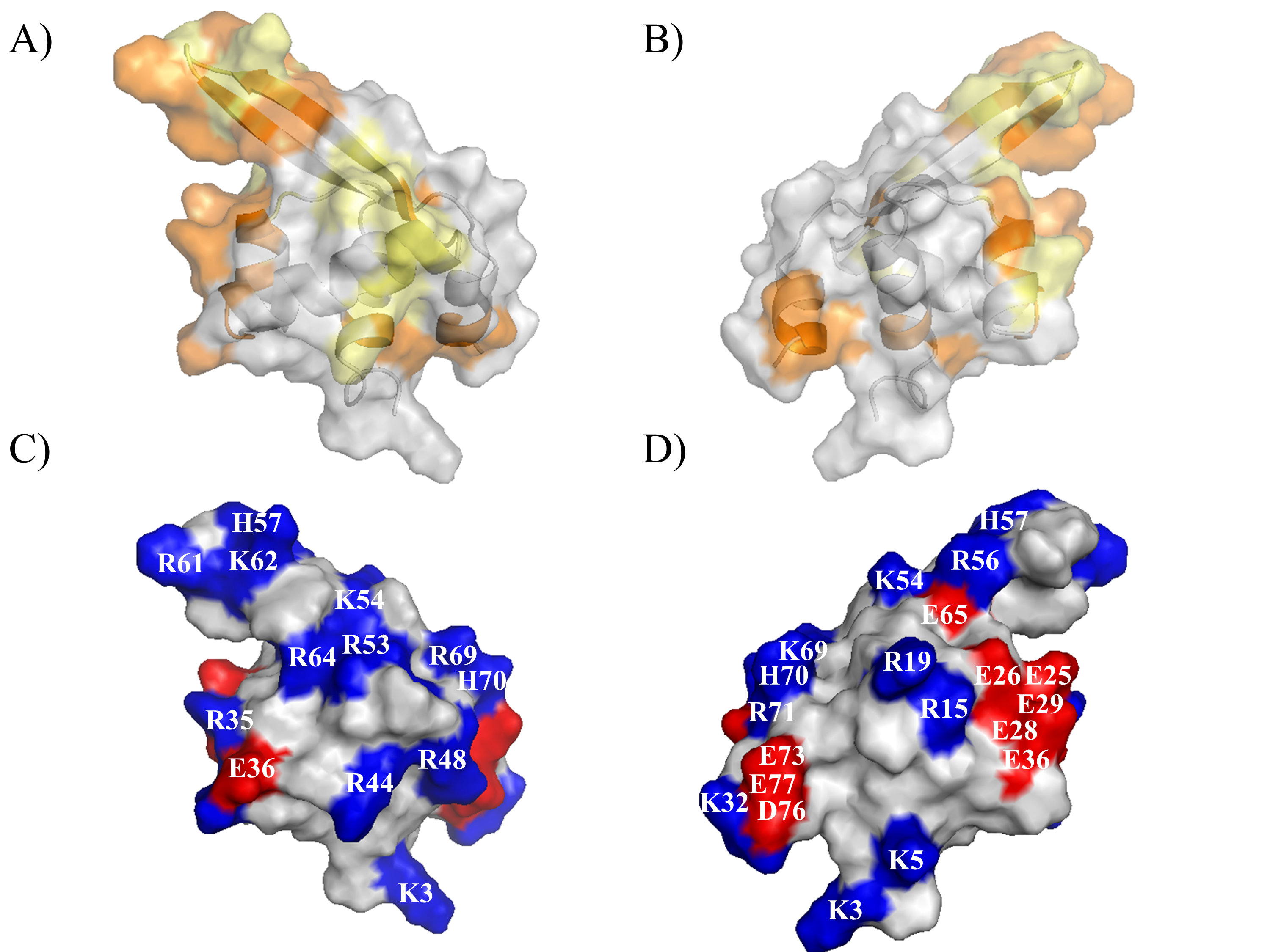


**Figure S7**.Comparison of the regions affected by the DNA interaction and the electrostatic surface potential maps of Stf76. (A-B) Ribbon and surface representation of Stf76 structure with mapping of the residues involved in DNA binding. Residues for which ΔHN_av_ are higher to mean and mean +SD values, are shown in gold and in orange, respectively. Residues with the most intensity reduction are shown in orange red. In (A) e (C) Stf76 is oriented to display the predicted DNA binding surface. (C-D) Positive and negative residues are colored in blue and in red, respectively, on the Stf76 surface. Neutral and hydrophobic residues are colored in gray. The figure was prepared using the PyMOL Molecular Graphics System, Version 1.5.0.4 Schrödinger, LLC.
